# Synthesis of 5-unsubstituted dihydropyrimidinone-4-carboxylates from deep eutectic mixtures

**DOI:** 10.3762/bjoc.18.37

**Published:** 2022-03-22

**Authors:** Sangram Gore, Sundarababu Baskaran, Burkhard König

**Affiliations:** 1 Department of Chemistry, Indian Institute of Technology Madras, Chennai 600036, India; 2 Institut für Organische Chemie, Universität Regensburg, Universitätsstraße 31, D-93040 Regensburg, Germany

**Keywords:** green protocol, mild conditions, no additional catalyst, solvent free, triple role of melt

## Abstract

A facile one-pot synthesis of 5-unsubstituted dihydropyrimidinones from β,γ-unsaturated ketoesters in low melting ʟ-(+)-tartaric acid–*N*,*N*-dimethylurea mixtures is reported. This solvent-free method is very general and provides easy access to 5-unsubstituted dihydropyrimidinone-4-carboxylate derivatives in good yields.

## Introduction

In recent years, dihydropyrimidinones (DHPMs) and their derivatives have attracted considerable attention due to the multifaceted pharmacological properties of this class of compounds [1–4]. The dihydropyrimidinone structure is found in calcium channel blockers [5–7], α1a adrenoceptor-selective antagonists [8–9], antihypertensive [10–13] and anti-inflammatory agents [14–15]. An interesting example is a rather simple DHPM derivative, monastrol, which specifically inhibits the motor activity of mitotic kinesin Eg5 and is therefore considered as a lead for the development of anticancer drugs [16].

**Figure 1:**
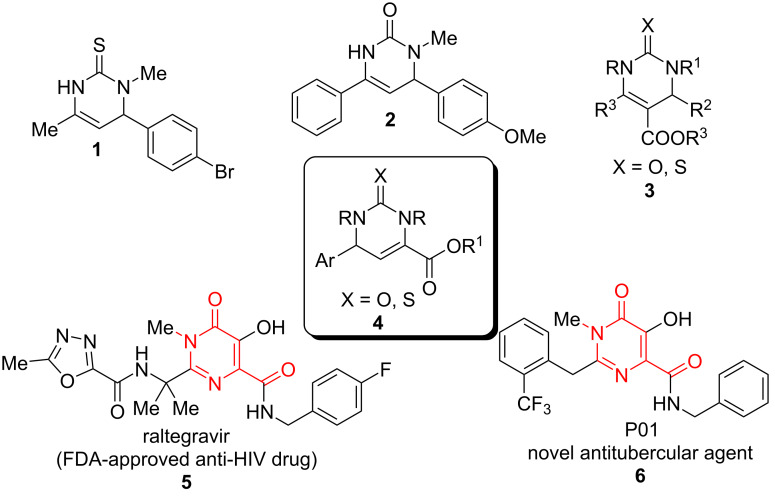
Biologically active functionalized DHPMs.

Of particular interest are 5-unsubstituted DHPMs [17], such as compounds **1** and **2**, which possess neuronal sodium channel blockade activities (Figure 1). Other examples are raltegravir, the first HIV-integrase inhibitor approved by the FDA for the treatment of HIV infection, derived from 5,6-dihydroxypyrimidine-4-carboxamide and *N*-methyl-4-hydroxypyrimidinone-carboxamide [18] and hydroxypyrimidinone carboxamide derivative P01, a potent inhibitor of *Mycobacterium tuberculosis* (Mtb) [19].

Owing to the biological significance of 5-unsubstituted dihydropyrimidinones, a variety of multistep protocols has been reported for the synthesis of 5-unsubstituted 3,4-dihydropyrimidin-2(1*H*)-ones [20–21]. Typically, dihydropyrimidinones are obtained via a Biginelli reaction leading to an ester group at C5 and an alkyl group at C6 position. A group of researchers from Merck reported the synthesis of 5-unsubstituted DHPMs via a Biginelli reaction followed by saponification of the ester and subsequent decarboxylation [22]. Later, Bussolari and McDonnell demonstrated the synthesis of 5-unsubstituted 3,4-dihydropyrimidinone-4-carboxylate derivatives by employing oxalacetic acid as a β-ketoester equivalent in the presence of TFA via a Biginelli reaction [23]. Lam and Fang reported the same synthesis under microwave conditions [24].

Very recently, Kambappa and co-workers reported a one-pot synthesis of 5-unsubstituted dihydropyrimidinone-4-carboxylate using gem-dibromomethylarene, oxalacetic acid, and urea [25]. Here the *gem*-dibromomethylarene moiety serves as an aldehyde equivalent. In addition, utilizing aromatic ketones as a β-ketoester equivalent, the synthesis of 5-unsubstituted DHPM bearing two aryl groups at the C4 and C6 positions was also reported [26]. Although several synthetic routes to 5-unsubstituted DHPM have been reported, there is still need for improvements in terms of higher yields, shorter reaction times, less hazardous or corrosive reagents, and fewer synthetic steps. Here, we report the facile and economic access to 5-unsubstituted DHPMs using a melt procedure avoiding organic solvents. The method is based on our previous reports of synthesis of organic molecules in the melt [27].

## Results and Discussion

Since 5-unsubstituted DHPMs bearing the carboxylic acid moiety at the C4 position allow versatile further functionalization and are biologically interesting DHPMs [17–21], we envisioned an environmentally benign cyclocondensation protocol using low melting mixtures as a green reaction medium. We have established low melting mixtures [28–31] based on carbohydrates, urea, and inorganic salts as an alternative to conventional solvents for carrying out a variety of organic transformations [32]. The stable melts are environmentally friendly as they are readily available from bulk renewable resources. Their simple production allows the replacement of organic solvents. The melts are stable against air and have very low vapor pressures resembling the properties of ionic liquids. In addition, the polarity of these melts is very high [33]. Recently, we have explored several organic transformations such as coupling reaction, cycloaddition reaction, synthesis of glycosylurea, dihydropyrimidinones, pyrimidopyrimidinediones, and functionalized indole derivatives in this novel and green reaction medium [34–38].

We have also developed an efficient method for the synthesis of trisubstituted hydantoin derivatives from β,γ-unsaturated ketoacids [39]. In the present study, in continuation of our interest in the synthesis of functionalized DHPMs [27], we utilized β,γ-unsaturated ketoesters and subjected them to the melt conditions to achieve the synthesis of 5-unsubstituted DHPMs.

We envisaged that the simple Michael addition reaction of urea derivatives with β,γ-unsaturated ketoesters and subsequent intramolecular condensation could lead to 5-unsubstituted DHPM derivatives. β,γ-Unsaturated ketoester **7**, derived from benzaldehyde and pyruvic acid [40], on exposure to ʟ-(+)-tartaric acid–*N*,*N*-dimethylurea (DMU) melt underwent smooth reaction to furnish the corresponding 5-unsubstituted dihydropyrimidinone-4-carboxylate derivative **8** in good yield (entry 1, Table 1). Encouraged by this observation, we tested the generality of this methodology by employing various electron-donating as well as electron-withdrawing groups on the aryl ring and the results are summarized in Table 1. The electron-rich (*E*)-ethyl 4-(4-methoxyphenyl)-2-oxobut-3-enoate (**11**) furnished the corresponding 5-unsubsituted DHPM derivative **12** on treatment with the ʟ-(+)-tartaric acid–DMU melt in very good yield (entry 3, Table 1). Moreover, electron-deficient β,γ-unsaturated ketoesters, such as (*E*)-ethyl 4-(4-nitrophenyl)-2-oxobut-3-enoate (**17**), afforded the corresponding 5-unsubstituted DHPM derivative **18** in good yield (entry 6, Table 1). Similarly, heteroaromatic aldehyde derived ketoester **21**, also underwent the tandem reaction to give the corresponding 5-unsubstituted DHPM derivative **22** in moderate yield (entry 9, Table 1). In addition to the aromatic part, the ester moiety of the β,γ-unsaturated ketoesters was also varied. (*E*)-Methyl 4-(2-azidophenyl)-2-oxobut-3-enoate (**23**), on exposure to the melt medium, yielded the corresponding 5-unsubstituted DHPM **24** (entry 10, Table 1) [41]. The scope of this method was further extended by employing this protocol to the synthesis of a thio derivative of 5-unsubstituted DHPM. Since thiourea does not form a clear melt with tartaric acid, the tartaric acid–choline chloride melt was employed for the reaction involving thiourea as one of the reactants. Hence, (*E*)-ethyl 4-(4-bromophenyl)-2-oxobut-3-enoate (**19**) on treatment with tartaric acid–choline chloride melt by employing thiourea as one of the reactants furnished the corresponding thio derivative of 5-unsubstituted DHPM derivative (entry 8, Table 1). The melt medium plays a triple role as solvent, catalyst and as reactant and furnishes the functionalized 5-unsubstituted dihydropyrimidinone-4-carboxylate derivatives.

**Table 1:** Synthesis of 5-unsubstituted DHPMs.^a^

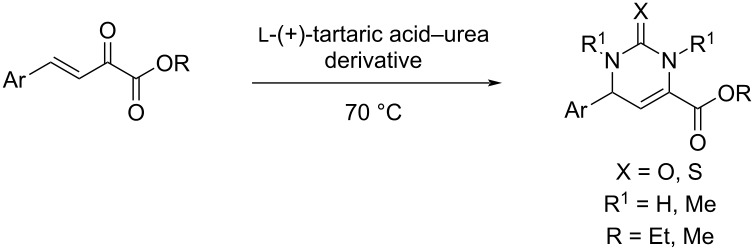				

Entry	Substrate	Time (h)	Product	Yield^b^ (%)

1	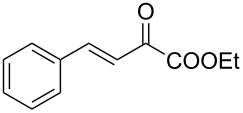 **7**	2	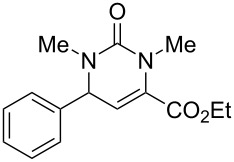 **8**	83
2	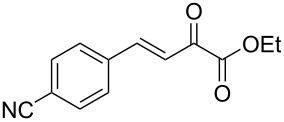 **9**	2.5	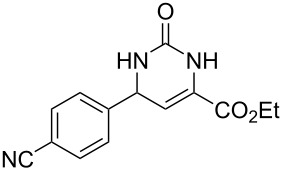 **10**	88^c^
3	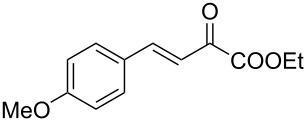 **11**	3	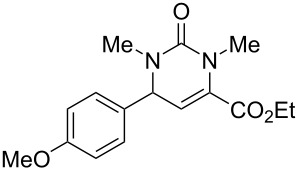 **12**	87
4	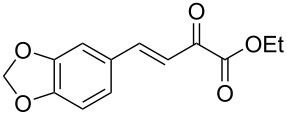 **13**	8	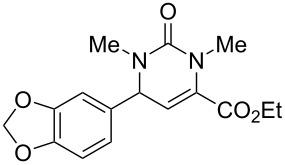 **14**	71
5	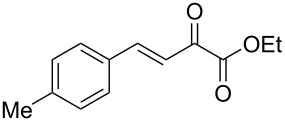 **15**	2.5	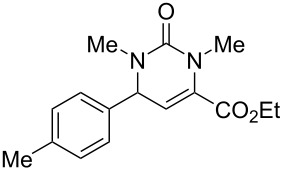 **16**	84
6	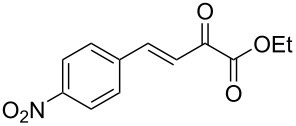 **17**	1.5	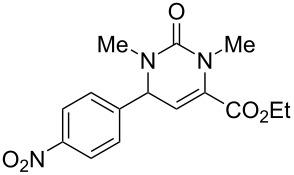 **18**	77
7	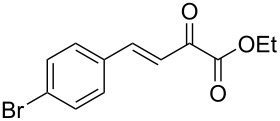 **19**	2	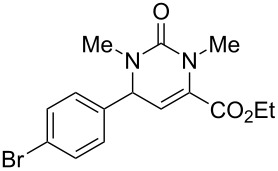 **20**	70
8	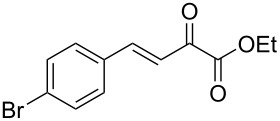 **19**	6	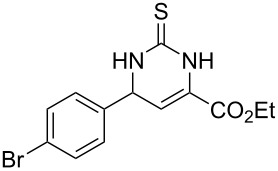 **20A**	48^d^
9	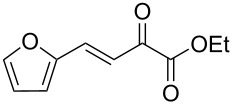 **21**	5	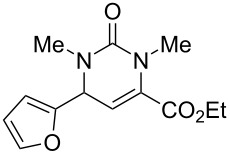 **22**	60
10	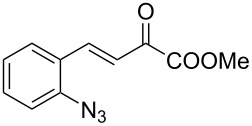 **23**	1.5	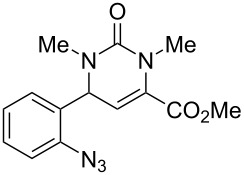 **24**	53
11	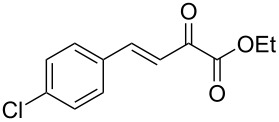 **25**	1.5	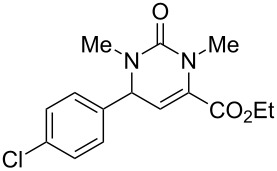 **26**	85
12	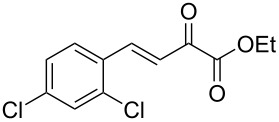 **27**	3.5	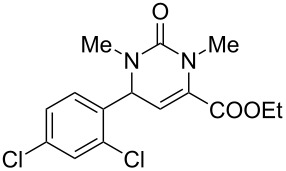 **28**	51

^a^β,γ-unsaturated ketoester (1 mmol) in ʟ-(+)-tartaric acid–DMU melt at 70 °C; ^b^isolated yield; ^c^at 90 °C in ʟ-(+)-tartaric acid–urea (2:3) melt; ^d^reaction carried out in the presence of thiourea in ʟ-(+)-tartaric acid–choline chloride melt (1:2) at 90 °C.

## Conclusion

In conclusion, a novel one-pot approach has been developed for the synthesis of 5-unsubstituted dihydropyrimidinone-4-carboxylate derivatives in good yields under environmentally benign conditions. Electron-rich as well as electron-deficient, highly functionalized β,γ-unsaturated ketoesters proved to be excellent substrates in this cyclocondensation reaction. The carboxylic ester substitution at C4 position provides the option for further chemical transformations on the DHPM skeleton. We hope that this environmentally benign one-pot method will find application in the synthesis of 5-unsubstituted dihydropyrimidinones.

## Supporting Information

File 1 Experimental procedures, characterization of products, copies of NMR spectra.
