# Supplementary material for: Synthesis of 5-unsubstituted dihydropyrimidinone-4-carboxylates from deep eutectic mixtures
Source: Beilstein J Org Chem. 2022 Mar 22;18:331–6. doi: 10.3762/bjoc.18.37 (PMC8965339; doi:10.3762/bjoc.18.37)
Supplement: File 1 — Experimental procedures, characterization of products, copies of NMR spectra. [file Beilstein_J_Org_Chem-18-331-s001.pdf]

BEILSTEIN JOURNAL OF ORGANIC CHEMISTRY

Supporting Information

for

Synthesis of 5-unsubstituted dihydropyrimidinone-4-carboxylates from deep eutectic mixtures

Sangram Gore, Sundarababu Baskaran and Burkhard König

Beilstein J. Org. Chem. **2022,** *18,* 331–336. doi:10.3762/bjoc.18.37

Experimental procedures, characterization of products, copies of NMR spectra

License and Terms: This is a supporting information file under the terms of the Creative Commons Attribution License (https://creativecommons.org/licenses/by/4.0). Please note that the reuse, redistribution and reproduction in particular requires that the author(s) and source are credited and that individual graphics may be subject to special legal provisions.
The license is subject to the *Beilstein Journal of Organic Chemistry* terms and conditions: (https://www.beilstein-journals.org/bjoc/terms)

Table of contents

General information S2

Preparation of β,γ-unsaturated ketoesters S2

Characterization data for β,γ-unsaturated ketoesters... S3–S6

General procedure for the synthesis of 5-unsubstituted dihydropyrimidinones S6

Characterization data for the 5-unsubstituted dihydropyrimidinones.................................. S7–S12

Copies of ^{1}H NMR and ^{13}C NMR spectra S13–S56

References S57

General information

^{1}H NMR spectra were recorded at 400 MHz, ^{13}C NMR spectra at 100 MHz. Chemical shifts are expressed in δ units relative to tetramethylsilane (TMS) as internal reference in DMSO-d_6, $CDCl_3$, or MeOD. FTIR spectra were recorded in $CHCl_3$ or neat. Column chromatography was performed on silica gel (60–120 mesh) using ethyl acetate and hexane as eluent.

Experimental section

Preparation of β,γ-unsaturated ketoesters:

To a stirred solution of aldehyde (10 mmol), pyruvic acid (10 mmol) in MeOH (15 mL) at 0 °C was added KOH (15 mmol) in MeOH (5 mL) dropwise over 10 minutes. The reaction mixture was allowed to warm to room temperature. After completion of the reaction monitored by TLC, the reaction mixture was filtered over a Buchner funnel. The residue was washed with cold MeOH (10 mL) and acidified with 1N HCl and extracted with EtOAc (10 mL × 3). The organic layer was dried over anhydrous Na_2SO_4 and concentrated under vacuum to give pure β,γ-unsaturated ketoacid in good yield.

To a stirred solution of β,γ-unsaturated ketoacid (10 mmol) in alcohol (25 mL) at 0 °C was added thionyl chloride (12 mmol) dropwise. The resultant reaction mixture was stirred at room temperature for 2 h. The reaction mixture was concentrated under vacuum and the crude reaction mass was quenched with saturated $NaHCO_3$ solution. The aqueous layer was extracted with ethyl acetate (10 mL × 3). The combined organic layer was dried over anhydrous Na_2SO_4 and concentrated under vacuum. The crude compound was purified using column chromatography over silica gel to give pure β,γ-unsaturated ketoester in good yield.

Characterization data for the β,γ-unsaturated ketoesters:

1. (*E*)-Ethyl 2-oxo-4-phenylbut-3-enoate (7) [1]:

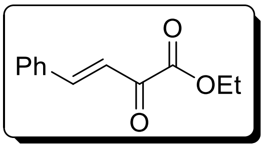

Yellow oil; Yield 80%; ^{1}H NMR (400 MHz, $CDCl_3$): δ 1.30 (t, 3H, J = 7.2 Hz), 4.29 (q, 2H, J = 6.8 Hz), 7.21–7.25 (m, 1H), 7.28–7.36 (m, 3H), 7.51 (d, 1H, J = 7.6 Hz), 7.74 (d, 1H, J = 16.4 Hz); ^{13}C NMR (100 MHz, $CDCl_3$): δ 14.1, 62.6, 120.7, 129.1, 129.2, 131.7, 134.1, 148.5, 162.3, 183.0; HRMS calcd. for $C_{12}H_{12}O_3Na$ (M^++Na) 227.0684, found 227.0694.

2. (*E*)-Ethyl 4-(4-cyanophenyl)-2-oxobut-3-enoate (9) [2]:

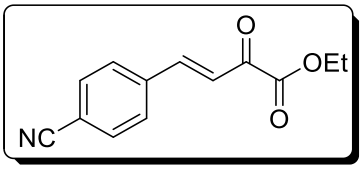

Yellow oil; Yield 78%; ^{1}H NMR (400 MHz, $CDCl_3$): δ 1.41 (t, 3H, J = 7.2 Hz), 4.40 (q, 2H, J = 7.2 Hz), 7.46 (d, 1H, J = 16.0 Hz), 7.72 (bs, 4H), 7.83 (d, 1H, J = 16.2 Hz); ^{13}C NMR (100 MHz, $CDCl_3$): δ 14.2, 63.0, 114.6, 118.3, 123.5, 129.3, 132.9, 138.3, 145.5, 161.7, 182.3.

3. (*E*)-Methyl 4-(2-azidophenyl)-2-oxobut-3-enoate (23):

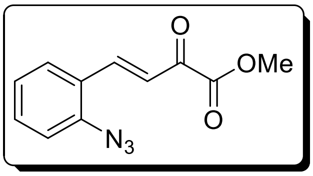

Yellow solid; Yield 51%; ^{1}H NMR (400 MHz, $CDCl_3$): δ 3.94 (s, 3H), 7.14–7.25 (m, 2H), 7.38 (d, 1H, J = 16.4 Hz), 7.44–7.55 (m, 1H), 7.69 (dd, 1H, J = 7.6, 0.8 Hz), 8.13 (d, 1H, J = 16.0 Hz); ^{13}C NMR (100 MHz, $CDCl_3$): δ 53.2, 119.1, 121.8, 125.1, 125.6, 128.8, 132.8, 140.6, 142.8, 162.7, 182.6; HRMS calcd. for $C_{11}H_{10}N_3O_3$ (M^++H) 232.0722, found 232.0712.

4. (*E*)-Ethyl 4-(4-methoxyphenyl)-2-oxobut-3-enoate (11) [1]:

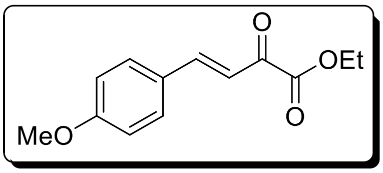

Yellow solid; Yield 84%; ^{1}H NMR (400 MHz, $CDCl_3$): δ 1.38 (dt, 3H, J = 7.2, 0.8 Hz), 3.82 (s, 3H), 4.36 (dq, 2H, J = 7.2, 0.8 Hz), 6.87–6.93 (m, 2H), 7.21 (dd, 1H, J = 16.0, 1.6 Hz), 7.56 (d, 2H, J = 7.6 Hz), 7.80 (d, 1H, J = 16.0 Hz); ^{13}C NMR (100 MHz, $CDCl_3$): δ 14.1, 55.5, 62.5, 114.6, 118.1, 126.8, 131.1, 148.4, 162.5, 162.6, 182.7; HRMS calcd. for $C_{13}H_{15}O_4$ (M^++H) 235.0970, found 235.0977.

5. (*E*)-Ethyl 4-(4-chlorophenyl)-2-oxobut-3-enoate (25) [1]:

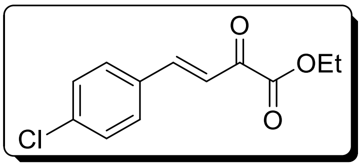

Yellow solid; Yield 73%; ^{1}H NMR (400 MHz, $CDCl_3$): δ 1.34 (t, 3H, J = 7.2 Hz), 4.32 (q, 2H, J = 7.2 Hz), 7.29–7.36 (m, 3H), 7.50 (d, 2H, J = 8.4 Hz), 7.74 (d, 1H, J = 16.0 Hz); ^{13}C NMR (100 MHz, $CDCl_3$): δ 14.2, 62.8, 121.0, 129.6, 130.3, 132.6, 137.8, 147.0, 162.1, 182.7; HRMS calcd. for $C_{12}H_{12}O_3Cl$ (M^++H) 239.0475, found 239.0477.

6. (*E*)-Ethyl 4-(benzo[*d*][1,3]dioxol-5-yl)-2-oxobut-3-enoate (13) [1]:

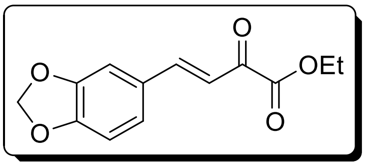

Yellow solid; Yield 48%; ^{1}H NMR (400 MHz, $CDCl_3$): δ 1.44 (t, 3H, J = 6.8 Hz), 4.42 (q, 2H, J = 7.2 Hz), 6.08 (s, 2H), 6.88 (d, 1H, J = 7.2 Hz), 7.15–7.20 (m, 2H), 7.27 (d, 1H, J = 17.2 Hz), 7.81 (d, 1H, J = 16.0 Hz); ^{13}C NMR (100 MHz, $CDCl_3$): δ 14.2, 62.6, 102.0, 107.0, 108.9, 118.6, 126.7, 128.7, 148.4, 148.7, 151.1, 162.5, 182.7; HRMS calcd. for $C_{13}H_{13}O_5$ (M^++H) 249.0763, found 249.0758.

7. (*E*)-Ethyl 2-oxo-4-*p*-tolylbut-3-enoate (15) [1]:

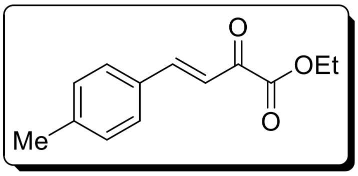

Yellow oil; Yield 88%; ^{1}H NMR (400 MHz, $CDCl_3$): δ 1.40 (t, 3H, J = 7.2 Hz), 2.38 (s, 3H), 4.38 (q, 2H, J = 6.8 Hz), 7.22 (d, 2H, J = 8.0 Hz), 7.31 (d, 1H, J = 16.4 Hz), 7.52 (d, 2H, J = 8.4 Hz), 7.83 (d, 1H, J = 16.4 Hz); ^{13}C NMR (100 MHz, $CDCl_3$): δ 14.2, 21.7, 62.5, 119.6, 129.2, 129.9, 131.4, 142.6, 148.7, 162.4, 183.0; HRMS calcd. for $C_{13}H_{14}O_3Na$ (M^++Na) 241.0841, found 241.0849.

8. (*E*)-Ethyl 4-(4-bromophenyl)-2-oxobut-3-enoate (19) [1]:

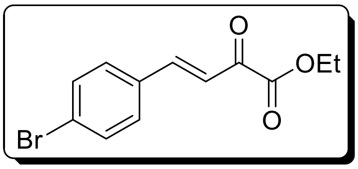

Yellow solid; Yield 69%; ^{1}H NMR (400 MHz, $CDCl_3$): δ 1.41 (t, 3H, J = 7.2 Hz), 4.39 (q, 2H, J = 6.8 Hz), 7.36 (d, 1H, J = 16.0 Hz), 7.49 (d, 2H, J = 8.4 Hz), 7.56 (d, 2H, J = 8.4 Hz), 7.79 (d, 1H, J = 16.4 Hz); ^{13}C NMR (100 MHz, $CDCl_3$): δ 14.2, 62.8, 121.0, 126.3, 130.4, 132.5, 133.0, 147.0, 162.1, 182.7; HRMS calcd. for $C_{12}H_{11}O_3BrNa$ (M^++Na) 304.9789, found 304.9800.

9. (*E*)-Ethyl 4-(4-nitrophenyl)-2-oxobut-3-enoate (17) [3]:

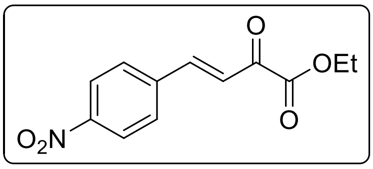

Yellow solid; Yield 75%; ^{1}H NMR (400 MHz, $CDCl_3$): δ 1.41 (t, 3H, J = 6.8 Hz), 4.40 (q, 2H, J = 7.2 Hz), 7.48 (d, 1H, J = 16.0 Hz), 7.62–7.82 (m, 2H), 7.87 (d, 1H, J = 16.0 Hz), 8.27 (d, 1H, J = 8.8 Hz); ^{13}C NMR (100 MHz, $CDCl_3$): δ 14.2, 63.0, 124.1, 124.4, 129.6, 140.1, 144.9, 149.2, 161.7, 182.3; HRMS calcd. for $C_{12}H_{11}NO_5Na$ (M^++Na) 272.0535, found 272.0525.

10. (*E*)-Ethyl 4-(furan-2-yl)-2-oxobut-3-enoate (21) [4]:

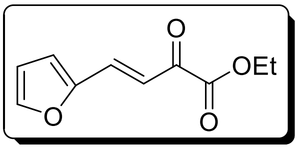

Yellow solid; Yield 65%; ^{1}H NMR (400 MHz, $CDCl_3$): δ 1.40 (t, 3H, J = 7.2 Hz), 4.37 (q, 2H, J = 7.2 Hz), 6.54 (dd, 1H, J = 2.8, 1.6 Hz), 6.82 (d, 1H, J = 3.6 Hz), 7.22 (d, 1H, J = 15.6 Hz), 7.55–7.65 (m, 2H); ^{13}C NMR (100 MHz, $CDCl_3$): δ 14.2, 62.6, 113.3, 118.2, 118.7, 133.7, 146.5, 151.2, 162.2, 182.5; HRMS calcd. for $C_{10}H_{10}O_4Na$ (M^++Na) 217.0477, found 217.0482.

11. (*E*)-Ethyl 4-(2,4-dichlorophenyl)-2-oxobut-3-enoate (27) [1]:

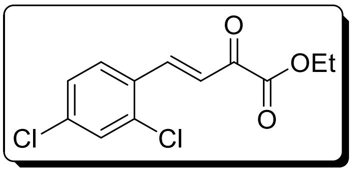

Yellow solid; Yield 45%; ^{1}H NMR (400 MHz, $CDCl_3$): δ 1.34 (t, 3H, J = 6.8 Hz), 4.32 (q, 2H, J = 6.8 Hz), 7.20–7.25 (m, 2H), 7.38 (d, 1H, J = 2.0 Hz), 8.11 (d, 1H, J = 16.4 Hz), 8.61 (d, 1H, J = 8.4 Hz); ^{13}C NMR (100 MHz, $CDCl_3$): δ 14.1, 62.8, 123.0, 127.8, 128.7, 130.3, 130.9, 136.8, 137.7, 142.5, 161.9, 182.5; HRMS calcd. for $C_{12}H_{11}O_3Cl_2$ (M^++H) 273.0085, found 273.0082.

General procedure for the formation of 5-unsubstituted dihydropyrimidinones:

Under conditions closely related to [5], 1.5 g of L-(+)-tartaric acid-DMU (30:70) mixture was heated to 70 °C to obtain a clear melt. To the melt, 1 mmol of β,γ-unsaturated ketoester was added at 70 °C. The reaction was monitored by thin layer chromatography. After the completion of reaction, the reaction was quenched by adding water while still hot. The reaction mixture was cooled to room temperature and the aqueous layer was extracted with DCM (3 × 5 mL) and washed with water (2 × 5 mL). The organic layer was dried over anhydrous Na_2SO_4 and concentrated under vacuum. The crude compound was purified using column chromatography over silica gel to afford pure 5-unsubtituted DHPM.

Characterization data for the 5-unsubstituted dihydropyrimidinones:

1. Ethyl 6-(4-cyanophenyl)-1,3-dimethyl-2-oxo-1,2,3,6-tetrahydropyrimidine-4-carboxylate (10)

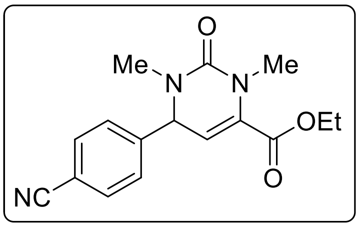

Colorless oil, Yield 88%; IR (neat): 2372, 1729, 1661, 1461, 1264 cm^{-1}; ^{1}H NMR (400 MHz, $CDCl_3$): δ 1.30 (t, 3H, J = 7.2 Hz), 2.82 (s, 3H), 3.25 (s, 3H) 4.20–4.27 (m, 2H), 4.98 (d, 1H, J = 5.6 Hz), 5.74 (d, 1H, J = 5.6 Hz), 7.35 (d, 2H, J = 8.4 Hz), 7.68 (d, 2H, J = 8.0 Hz); ^{13}C NMR (100 MHz, $CDCl_3$): δ 14.2, 33.1, 34.4, 61.8, 62.0, 109.7, 112.5, 118.5, 127.3, 132.9, 133.2, 145.3, 154.5, 162.4; HRMS calcd. for $C_{16}H_{18}N_3O_3$ (M^{+}+H) 300.1348, found 300.1362.

2. Ethyl 1,3-dimethyl-6-(4-nitrophenyl)-2-oxo-1,2,3,6-tetrahydropyrimidine-4-carboxylate (18)

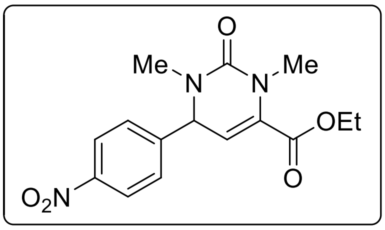

Pale yellow oil; Yield 77%; IR (neat): 2932, 1727, 1661, 1523, 1255, 1090 cm^{-1}; ^{1}H NMR (400 MHz, $CDCl_3$): δ 1.30 (t, 3H, J = 6.8 Hz), 2.84 (s, 3H), 3.26 (s, 3H), 4.19–4.29 (m, 2H), 5.05 (d, 1H, J = 5.6 Hz), 5.75 (d, 1H, J = 5.6 Hz), 7.38–7.43 (m, 2H), 8.22–8.27 (m, 2H); ^{13}C NMR (100 MHz, $CDCl_3$): δ 14.2, 33.1, 34.5, 61.6, 62.0, 109.5, 124.7, 127.4, 133.1, 147.2, 148.0, 154.4, 162.4. HRMS calcd. for $C_{15}H_{17}N_3O_5Na$ (M^{+}+Na) 342.1066, found 342.1078.

3. Ethyl 6-(furan-2-yl)-1,3-dimethyl-2-oxo-1,2,3,6-tetrahydropyrimidine-4-carboxylate (22)

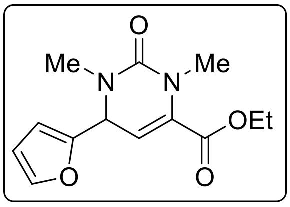

Colorless oil; Yield 60%; IR (neat): 1730, 1660, 1423, 1265, 1092 cm^{-1}; ^{1}H NMR (400 MHz, $CDCl_3$): δ 1.32 (t, 3H, J = 7.2 Hz), 2.90 (s, 3H), 3.22 (s, 3H), 4.20–4.31 (m, 2H), 4.92 (d, 1H, J = 6.0 Hz), 5.87 (d, 1H, J = 6.0 Hz), 6.23 (d, 1H, J = 3.2 Hz), 6.32 (dd, 1H, J = 3.2, 2.0 Hz), 7.37 (t, 1H, J =1.2 Hz); ^{13}C NMR (100 MHz, $CDCl_3$): δ 14.1, 33.0, 34.1, 54.6, 61.7, 107.8, 108.7, 110.4, 133.8, 143.1, 151.6, 154.9, 162.5. HRMS calcd. for $C_{13}H_{16}N_2O_4Na$ (M^++Na) 287.1008, found 287.1008.

4. Ethyl 6-(4-chlorophenyl)-1,3-dimethyl-2-oxo-1,2,3,6-tetrahydropyrimidine-4-carboxylate (26)

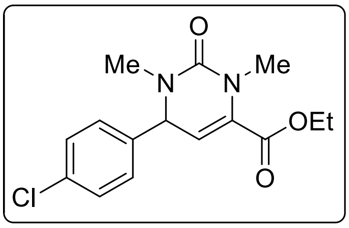

Colorless oil; Yield 85%; IR (neat): 1729, 1661, 1423, 1265, 1091 cm^{-1}; ^{1}H NMR (400 MHz, $CDCl_3$): δ 1.29 (t, 3H, J = 7.2 Hz), 2.80 (s, 3H), 3.25 (s, 3H), 4.17–4.30 (m, 2H), 4.89 (d, 1H, J = 5.6 Hz), 5.76 (d, 1H, J = 5.6 Hz), 7.14–7.18 (m, 2H), 7.32–7.36 (m, 2H); ^{13}C NMR (100 MHz, $CDCl_3$): δ 14.2, 33.1, 34.2, 61.5, 61.8, 111.0, 128.0, 129.5, 132.3, 134.4, 138.6, 154.6, 162.6. HRMS calcd. for $C_{15}H_{18}N_2O_3Cl$ (M^++H) 309.1066, found 309.1009.

5. Ethyl 1,3-dimethyl-2-oxo-6-phenyl-1,2,3,6-tetrahydropyrimidine-4-carboxylate (8)

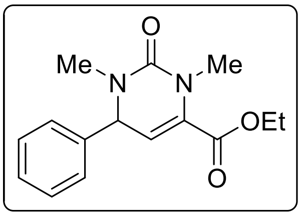

Colorless oil; Yield 83%; IR (neat): 2925, 1715, 1664, 1455, 1248[1]; ^{1}H NMR (400 MHz, $CDCl_3$): δ 1.30 (t, 3H, J = 7.2 Hz), 2.82 (s, 3H), 3.27 (s, 3H), 4.17–4.30 (m, 2H), 4.91 (d, 1H, J = 5.6 Hz), 5.83 (d, 1H, J = 5.6 Hz), 7.24 (d, 2H, J = 6.8 Hz), 7.30–7.41 (m, 3H); ^{13}C NMR (100 MHz, $CDCl_3$): δ 14.2, 33.1, 34.2, 61.7, 62.1, 111.8, 126.7, 128.6, 129.3, 132.0, 140.0, 154.8, 162.7; HRMS calcd. for $C_{15}H_{19}N_2O_3$ (M^++H) 275.1396, found 275.1405.

6. Ethyl 1,3-dimethyl-2-oxo-6-*p*-tolyl-1,2,3,6-tetrahydropyrimidine-4-carboxylate (16)

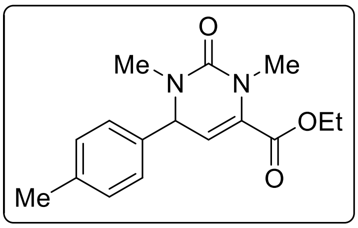

Colorless oil; Yield 84%; IR (neat): 2923, 1727, 1661, 1460, 1086 cm^{-1}; ^{1}H NMR (400 MHz, $CDCl_3$): δ 1.28 (t, 3H, J = 6.8 Hz), 2.34 (s, 3H), 2.80 (s, 3H), 3.25 (s, 3H), 4.15–4.28 (m, 2H), 4.86 (d, 1H, J = 5.6 Hz), 5.81 (d, 1H, J = 5.6 Hz), 7.11 (d, 2H, J = 8.0 Hz), 7.17 (d, 2H, J = 8.0 Hz); ^{13}C NMR (100 MHz, $CDCl_3$): δ 14.2, 21.2, 33.0, 34.1, 61.6, 61.8, 112.1, 126.6, 129.9, 131.9, 137.1, 138.4, 154.8, 162.8; HRMS calcd. for $C_{16}H_{21}N_2O_3$ (M^++H) 289.1552, found 289.1552.

7. Ethyl 6-(4-bromophenyl)-1,3-dimethyl-2-thioxo-1,2,3,6-tetrahydropyrimidine-4-carboxylate (20A)

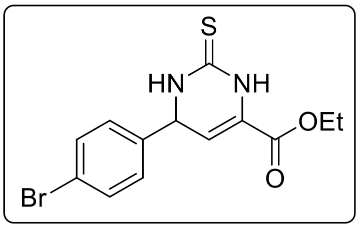

Yellow semisolid; Yield 48%; IR (neat): 1735, 1657, 1586, 1266, 1072 cm^{-1}; ^{1}H NMR (400 MHz, $CDCl_3$): δ 1.32 (t, 3H, J = 6.8 Hz), 4.27 (q, 2H, J = 4.6 Hz), 4.80 (d, 1H, J = 6.2 Hz), 6.34 (d, 1H, J = 6.0 Hz), 7.13–7.18 (m, 2H), 7.43–7.48 (m, 2H); ^{13}C NMR (100 MHz, $CDCl_3$): δ 14.4, 43.0, 61.5, 110.9, 122.5, 129.4, 132.3, 139.2, 140.5, 155.3, 165.3. HRMS calcd. for $C_{13}H_{14}N_2O_2SBr$ (M^++H) 340.9959, found 340.9974.

8. Ethyl 6-(4-bromophenyl)-1,3-dimethyl-2-oxo-1,2,3,6-tetrahydropyrimidine-4-carboxylate (20)

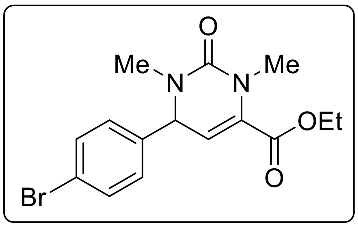

Colorless oil; Yield 70%; IR (neat): 2926 1727, 1662, 1478, 1260 cm^{-1}; ^{1}H NMR (400 MHz, $CDCl_3$): δ 1.28 (t, 3H, J = 7.2 Hz), 2.79 (s, 3H), 3.23 (s, 3H), 4.16–4.29 (m, 2H), 4.87 (d, 1H, J = 5.2 Hz), 5.75 (d, 1H, J = 5.2 Hz), 7.09 (d, 1H, J = 8.4 Hz), 7.48 (d, 1H, J = 8.4 Hz); ^{13}C NMR (100 MHz, $CDCl_3$): δ 14.1, 33.0, 34.1, 61.5, 61.8, 110.9, 122.5, 128.3, 132.3, 132.4, 139.1, 154.5, 162.5. HRMS calcd. for $C_{15}H_{18}N_2O_3Br_1$ (M^++H) 353.0501, found 353.0500.

9. Ethyl 6-(benzo[*d*][1,3]dioxol-5-yl)-1,3-dimethyl-2-oxo-1,2,3,6-tetrahydropyrimidine-4-carboxylate (14)

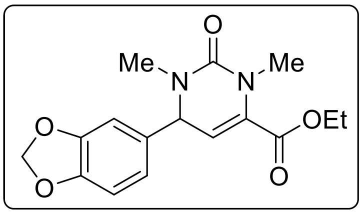

Colorless oil; Yield 71%; IR (neat): 2985, 1726, 1658, 1265 cm^{-1}; ^{1}H NMR (400 MHz, $CDCl_3$): δ 1.29 (t, 3H, J = 7.2 Hz), 2.79 (s, 3H), 3.24 (s, 3H), 4.18–4.28 (m, 2H), 4.80 (d, 1H, J = 5.2 Hz), 5.77 (d, 1H, J = 5.6 Hz), 5.96 (s, 2H), 6.68 (ddd, 2H, J = 9.6,1.6 Hz), 6.76 (d, 1H, J = 7.6 Hz); ^{13}C NMR (100 MHz, $CDCl_3$): δ 14.2, 33.0, 34.0, 61.7, 61.9, 101.4, 107.1, 108.6, 111.7, 120.2, 132.0, 134.0, 147.9, 148.6, 154.6, 162.7. HRMS calcd. for $C_{16}H_{19}N_2O_5$ (M^++H) 319.1294, found 319.1304.

10. Ethyl 6-(4-methoxyphenyl)-1,3-dimethyl-2-oxo-1,2,3,6-tetrahydropyrimidine-4-carboxylate (12)

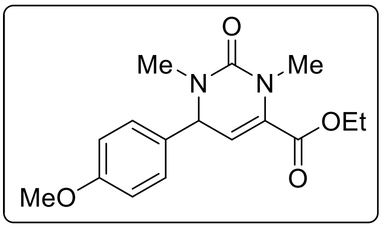

Colorless oil; Yield 87%; IR (neat): 1725, 1646, 1464, 1255 cm^{-1}; ^{1}H NMR (400 MHz, $CDCl_3$): δ 1.29 (t, 3H, J = 7.2 Hz), 2.79 (s, 3H), 3.25 (s, 3H), 3.80 (s, 3H), 4.16–4.29 (m, 2H), 4.84 (d, 1H, J = 5.6 Hz), 5.80 (d, 1H, J = 5.6 Hz), 6.86–6.91 (m, 1H), 7.12–7.18 (m, 2H); ^{13}C NMR (100 MHz, $CDCl_3$): δ 14.2, 33.1, 34.0, 55.5, 61.5, 61.7, 112.2, 114.5, 128.0, 131.8, 132.1, 154.7, 159.8, 162.8; HRMS calcd. for $C_{16}H_{21}N_2O_4$ (M^++H) 305.1501, found 305.1496.

11. Ethyl 6-(2-azidophenyl)-1,3-dimethyl-2-oxo-1,2,3,6-tetrahydropyrimidine-4-carboxylate (24)

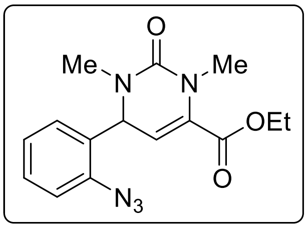

Colorless oil; Yield 53%; IR (neat): 2924, 2124, 1728, 1663, 1260 cm^{-1}; ^{1}H NMR (400 MHz, $CDCl_3$): δ 2.83 (s, 3H), 3.23 (s, 3H), 3.77 (s, 3H), 5.24 (d, 1H, J = 6.0 Hz), 5.93 (d, 1H, J = 6.0 Hz), 7.13–7.20 (m, 3H,), 7.31–7.37 (m, 1H); ^{13}C NMR (100 MHz, $CDCl_3$): δ 33.0, 34.4, 52.5, 56.6, 111.3, 118.6, 125.8, 127.3, 129.5, 130.7, 132.1, 136.7, 155.4, 163.1. HRMS calcd. for $C_{14}H_{16}N_5O_3$ (M^++H) 302.1253, found 302.1243.

12. Ethyl 6-(2,4-dichlorophenyl)-1,3-dimethyl-2-oxo-1,2,3,6-tetrahydropyrimidine-4-carboxylate (28)

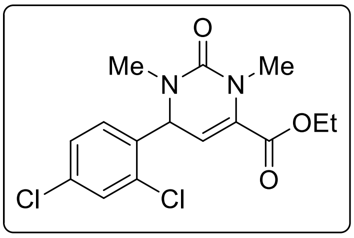

Colorless oil; Yield 51%; IR (neat): 2928, 1729, 1668, 1468, 1255, 1090 cm^{-1}; ^{1}H NMR (400 MHz, $CDCl_3$): δ 1.23 (t, 3H, J = 7.2 Hz), 2.78 (s, 3H), 3.17 (s, 3H), 4.10–4.24 (m, 2H), 5.32 (d, 1H, J = 6.0 Hz), 5.82 (d, 1H, J = 6.0 Hz), 7.07 (d, 1H, J = 8.0 Hz), 7.21 (dd, 1H, J = 8.4, 2.0 Hz), 7.34 (d, 1H, J = 2.0 Hz); ^{13}C NMR (100 MHz, $CDCl_3$): δ 14.2, 33.0, 34.4, 58.3, 61.9, 109.1, 128.4, 130.0, 132.5, 133.0, 134.7, 135.9, 155.0, 162.5. HRMS calcd. for $C_{15}H_{16}Cl_2N_2O_3Na$ (M^++Na) 365.0436, found 365.0448.

Copies of ^{1}H NMR and ^{13}C NMR spectra

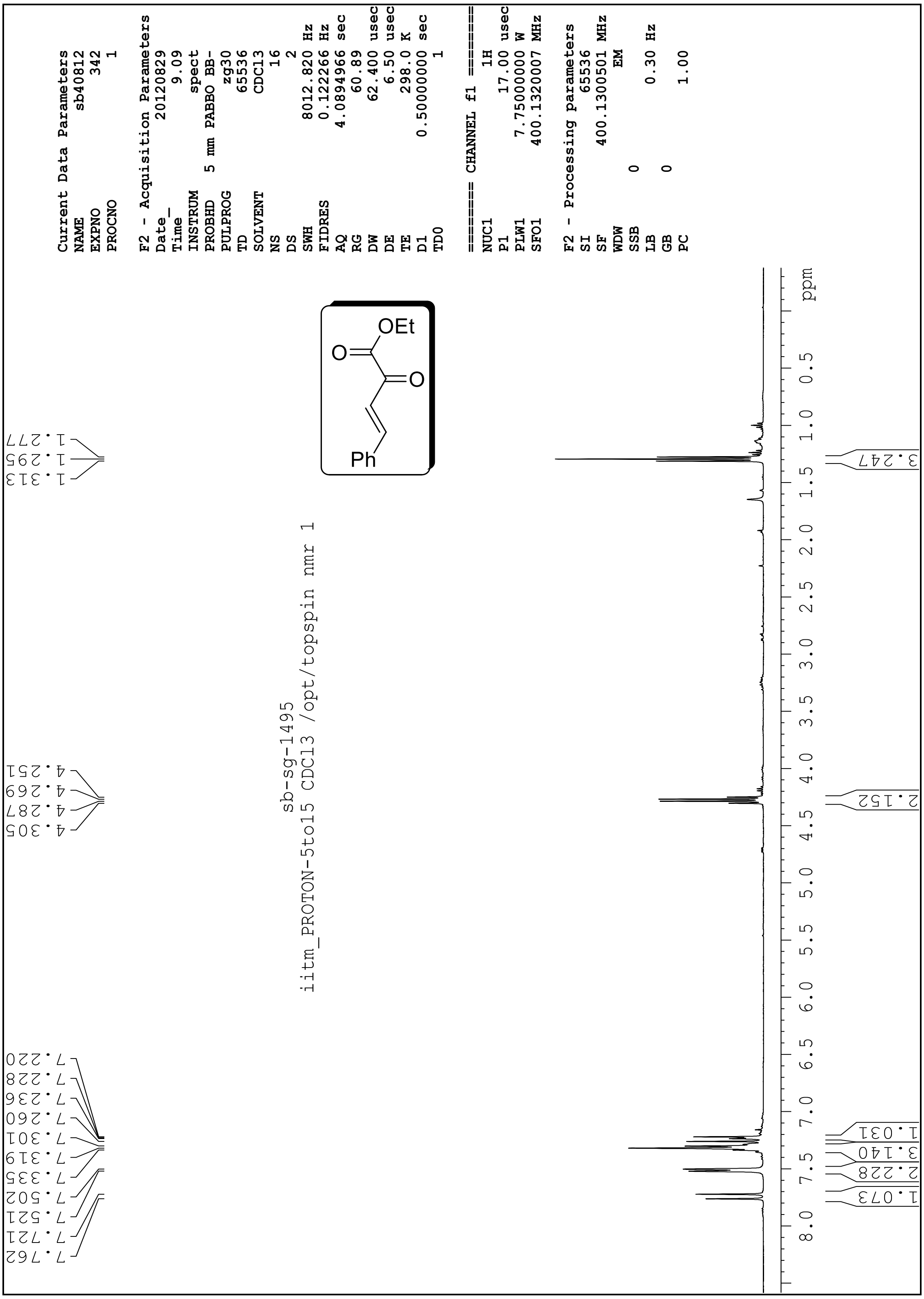

^{1}H NMR spectrum of compound **7**

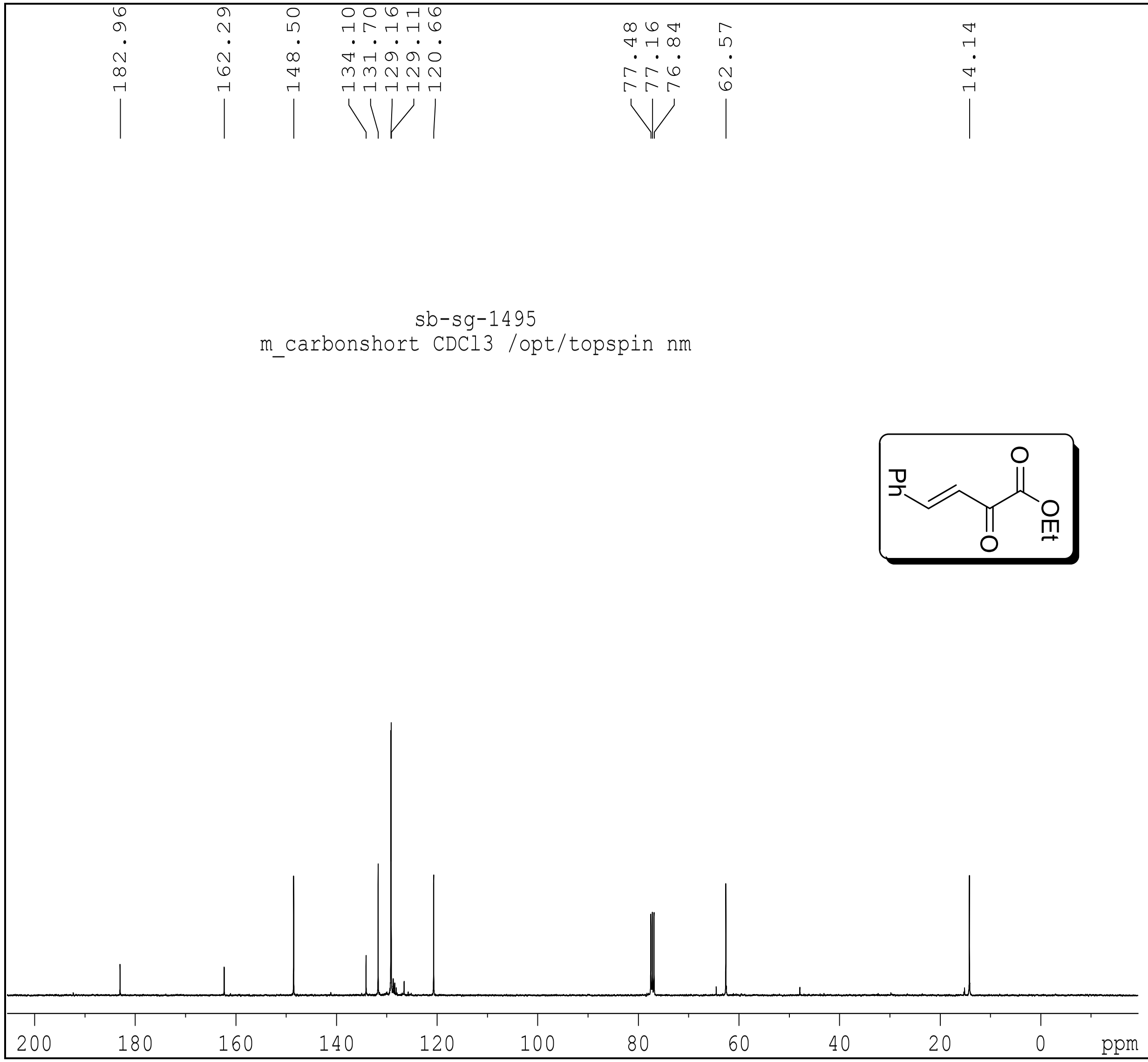


```
Current Data Parameters
NAME              sb40812
EXPNO                 343
PROCNO                  1

F2 - Acquisition Parameters
Date_            20120829
Time                 9.32
INSTRUM             spect
PROBHD   5 mm PABBO BB-
PULPROG           zgpg30
TD                  16540
SOLVENT             CDCl3
NS                    900
DS                      4
SWH             24038.461 Hz
FIDRES           1.453353 Hz
AQ              0.3440820 sec
RG                 200.34
DW                 20.800 usec
DE                   6.50 usec
TE                  298.0 K
D1             1.00000000 sec
D11            0.03000000 sec
TD0                     1

======== CHANNEL f1 ========
NUC1                  13C
P1                   9.25 usec
PLW1          47.00000000 W
SFO1         100.6228289 MHz

======== CHANNEL f2 ========
CPDPRG2           waltz16
NUC2                   1H
PCPD2               90.00 usec
PLW2           7.75000000 W
PLW12          0.27651000 W
PLW13          0.22397999 W
SFO2         400.1316005 MHz

F2 - Processing parameters
SI                  32768
SF           100.6127622 MHz
WDW                    EM
SSB      0
LB                   3.00 Hz
GB       0
PC                   1.40
```

^{13}C NMR spectrum of compound **7**

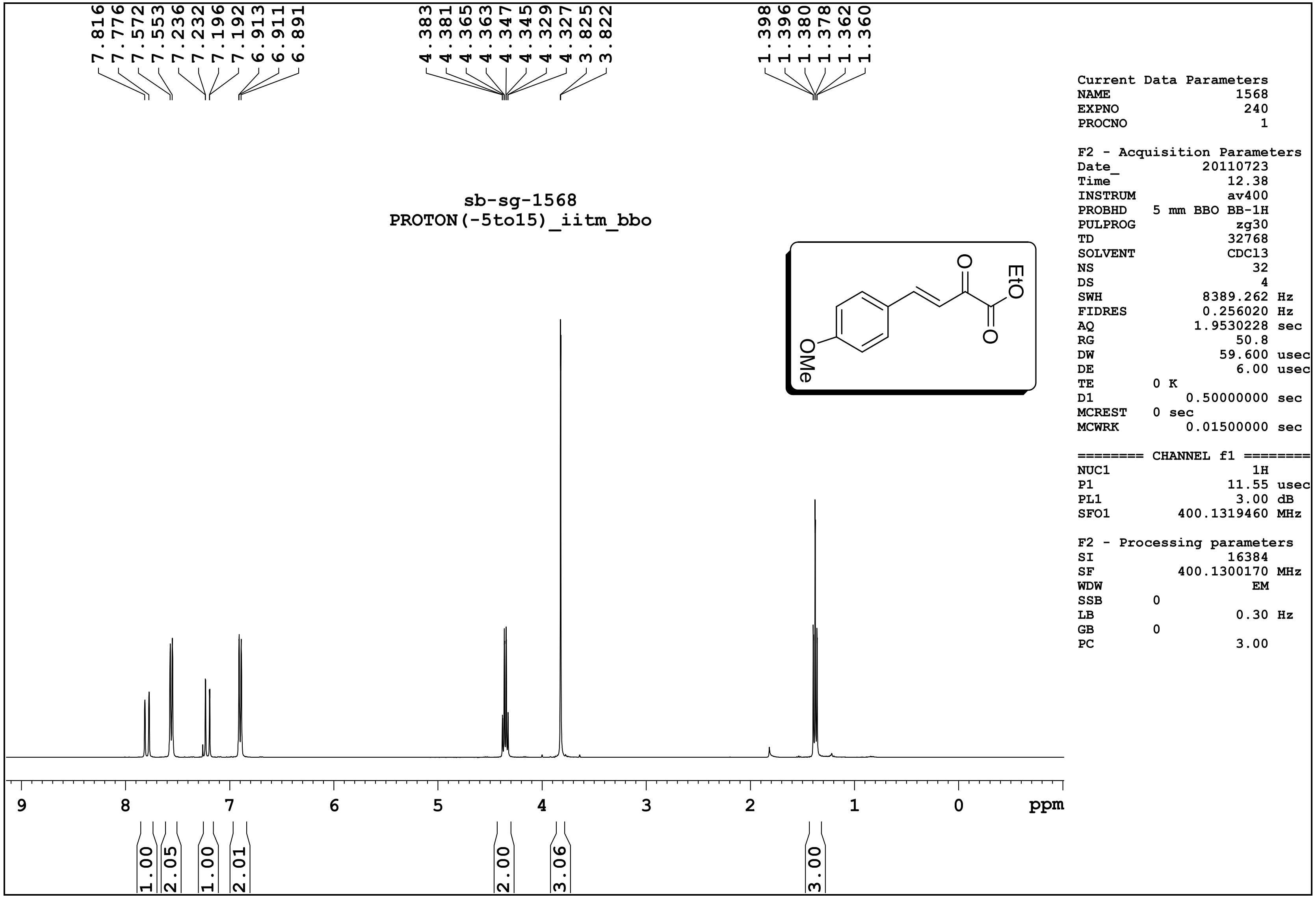

^{1}H NMR spectrum of compound **11**

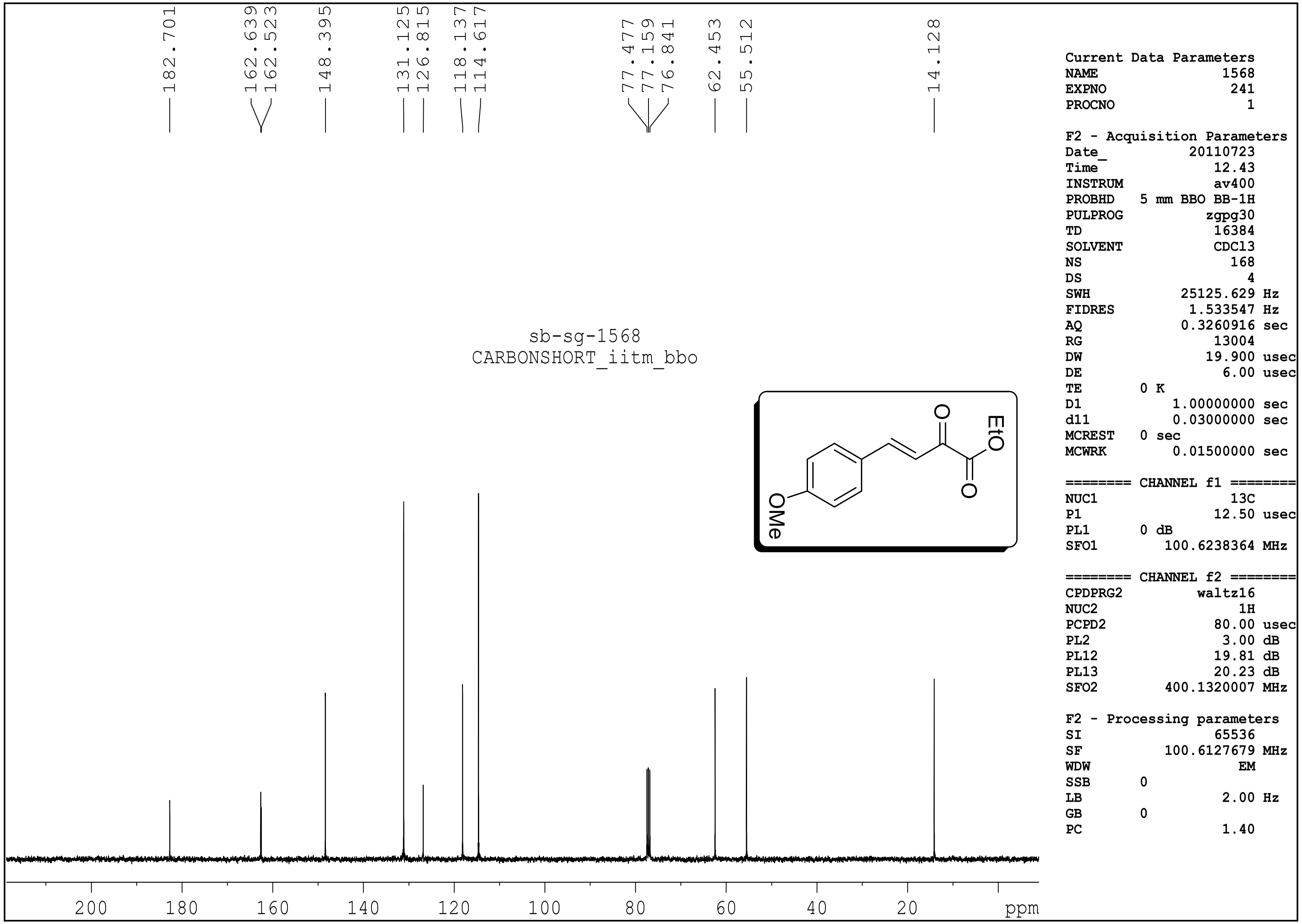

^{13}C NMR spectrum of compound **11**

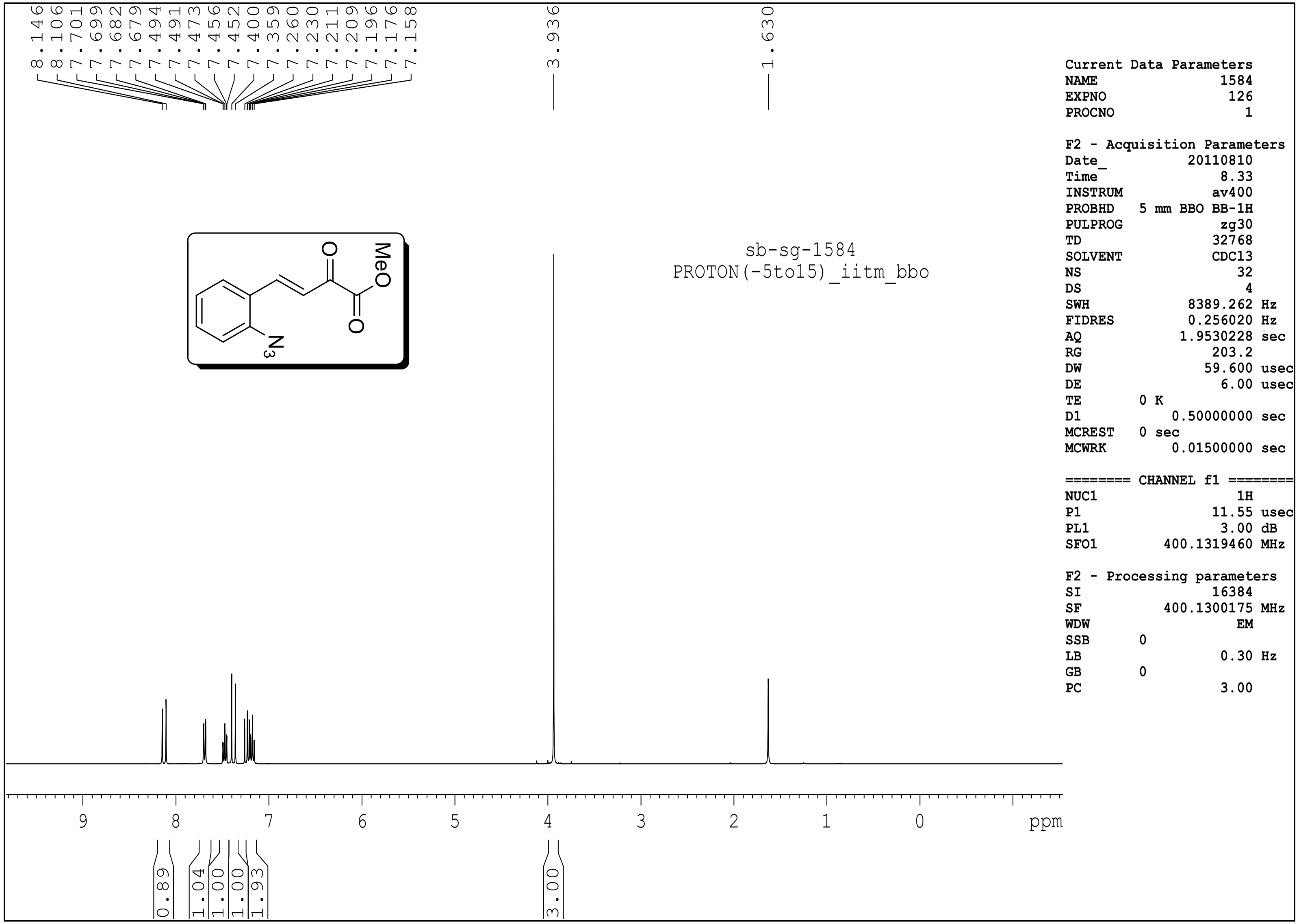

^{1}H NMR spectrum of compound **23**

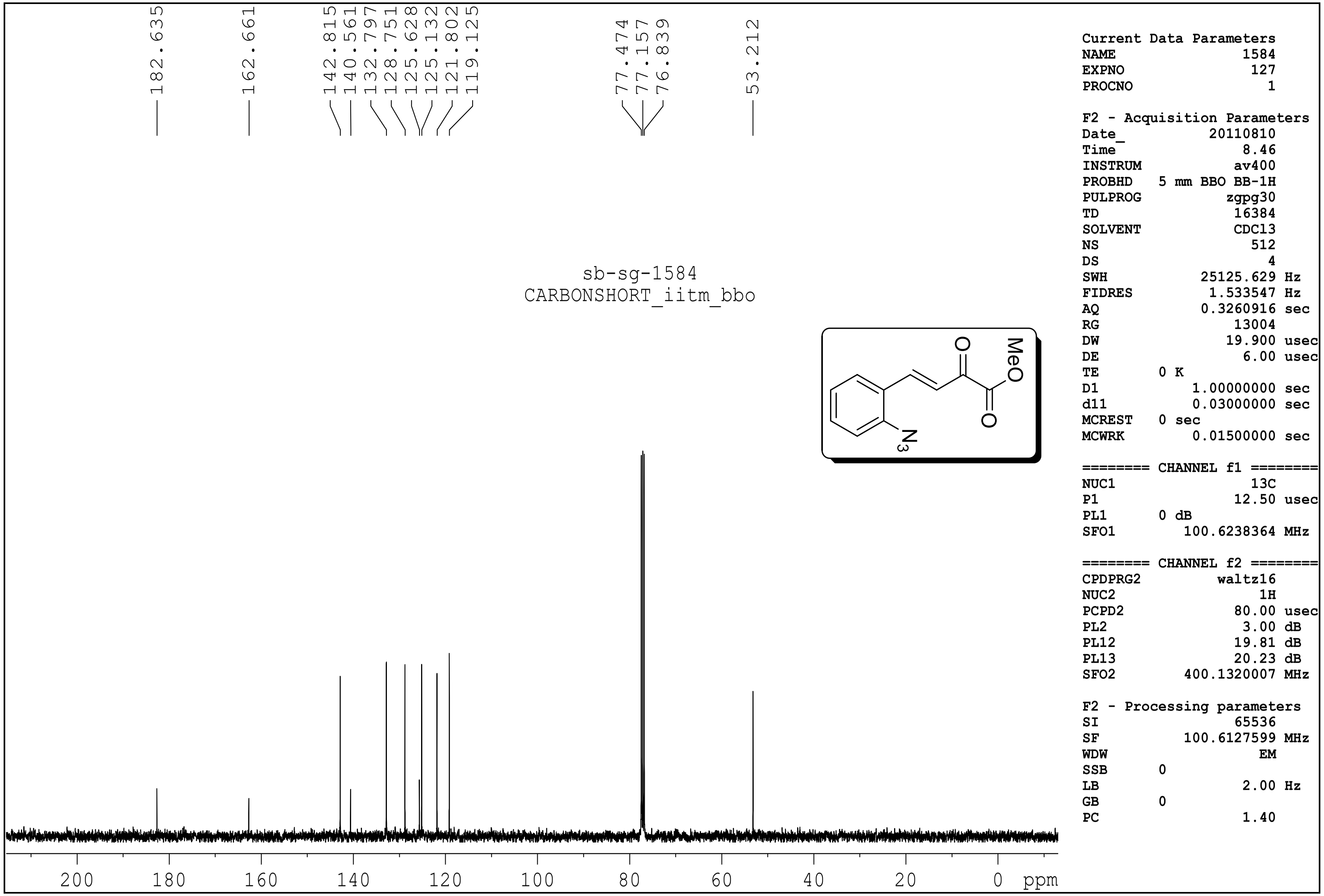

^{13}C NMR spectrum of compound **23**

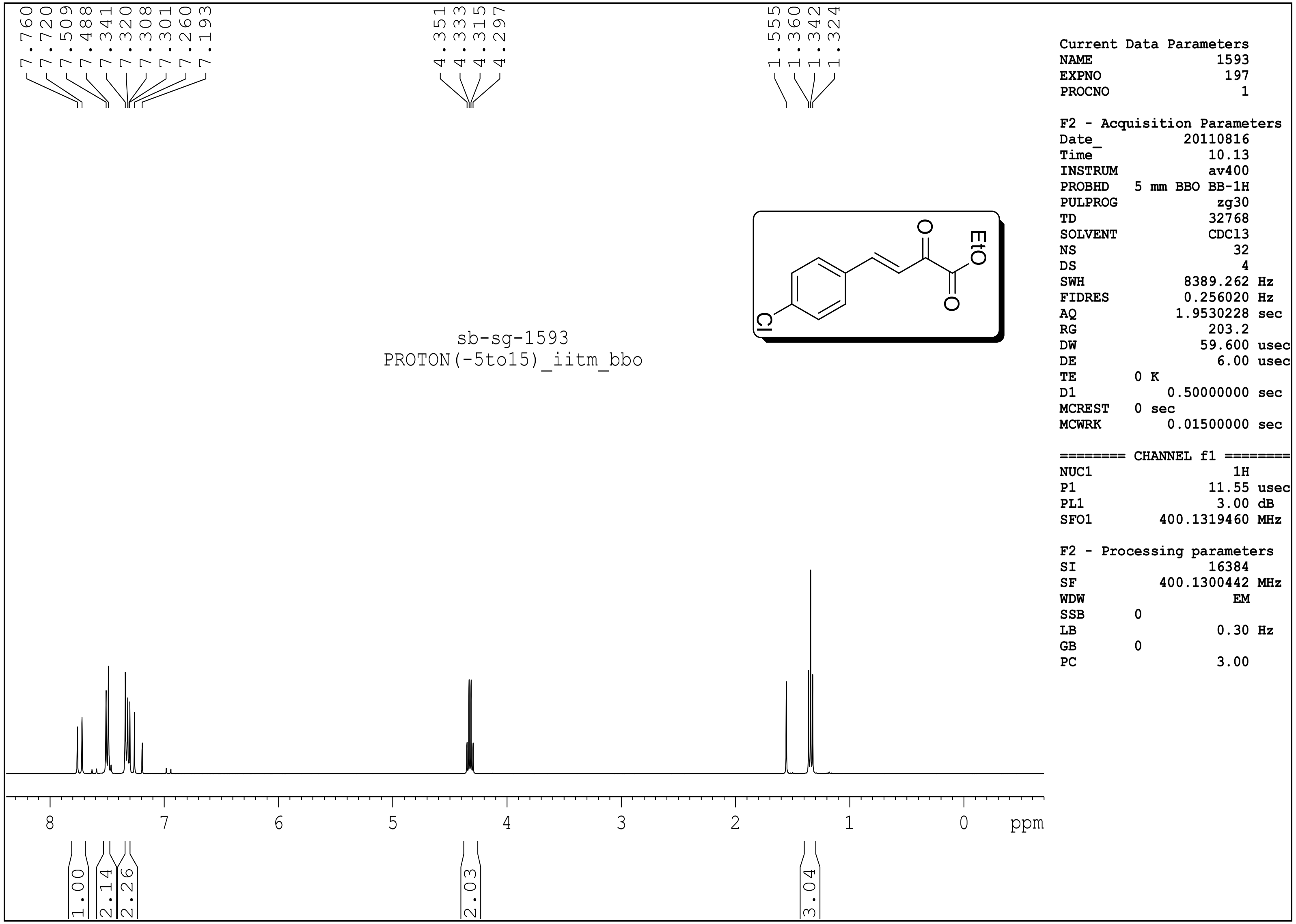

^{1}H NMR spectrum of compound **25**

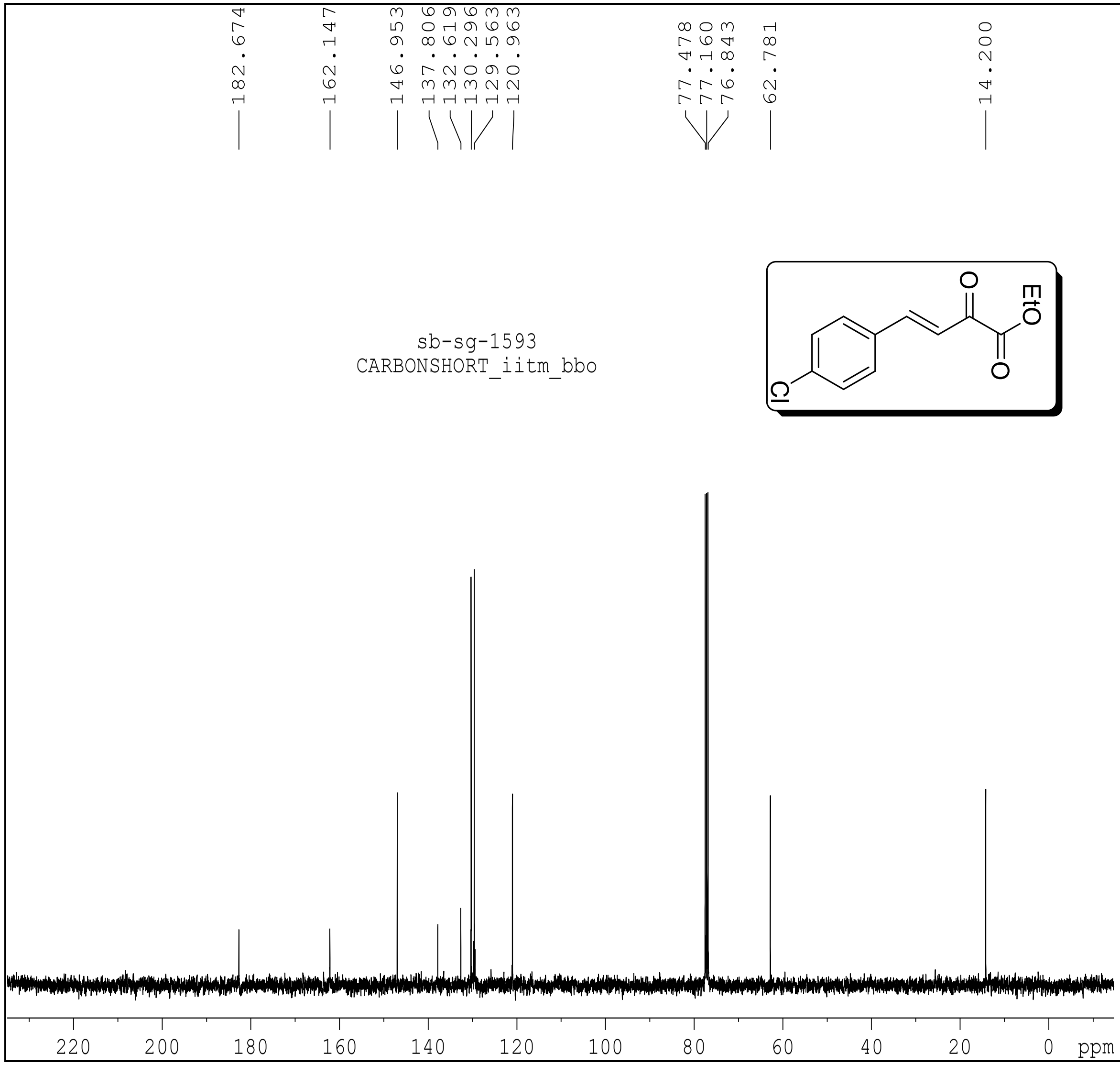


```
Current Data Parameters
NAME                 1593
EXPNO                 198
PROCNO                  1

F2 - Acquisition Parameters
Date_            20110816
Time                10.20
INSTRUM             av400
PROBHD   5 mm BBO BB-1H
PULPROG           zgpg30
TD                  16384
SOLVENT             CDCl3
NS                    389
DS                      4
SWH           25125.629 Hz
FIDRES         1.533547 Hz
AQ            0.3260916 sec
RG                  13004
DW                 19.900 usec
DE                   6.00 usec
TE        0 K
D1           1.00000000 sec
d11          0.03000000 sec
MCREST    0 sec
MCWRK        0.01500000 sec

======== CHANNEL f1 ========
NUC1                  13C
P1                  12.50 usec
PL1       0 dB
SFO1       100.6238364 MHz

======== CHANNEL f2 ========
CPDPRG2           waltz16
NUC2                   1H
PCPD2               80.00 usec
PL2                  3.00 dB
PL12                19.81 dB
PL13                20.23 dB
SFO2       400.1320007 MHz

F2 - Processing parameters
SI                  65536
SF         100.6127595 MHz
WDW                    EM
SSB       0
LB                   2.00 Hz
GB        0
PC                   1.40
```

^{13}C NMR spectrum of compound **25**

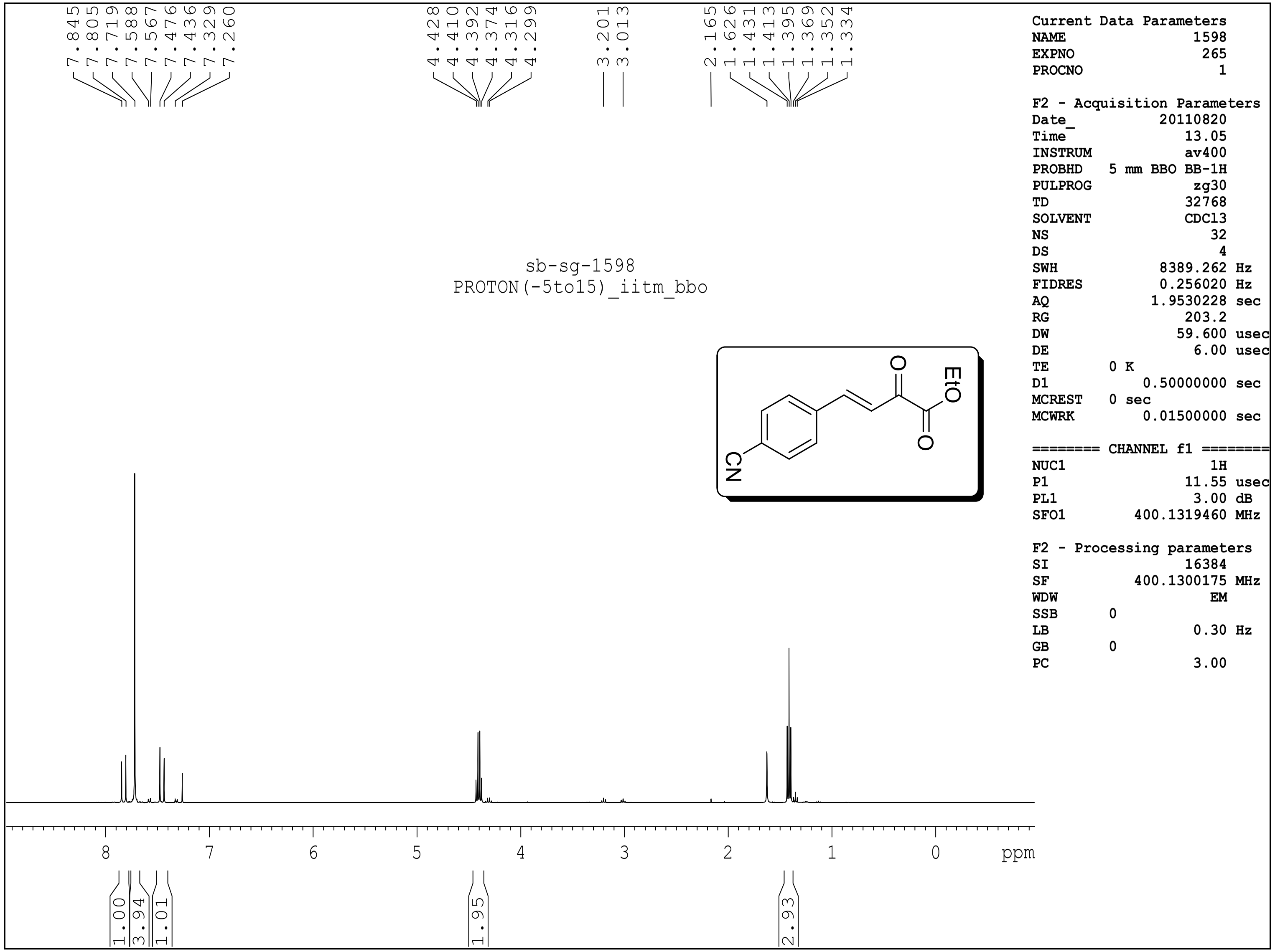

^{1}H NMR spectrum of compound **9**

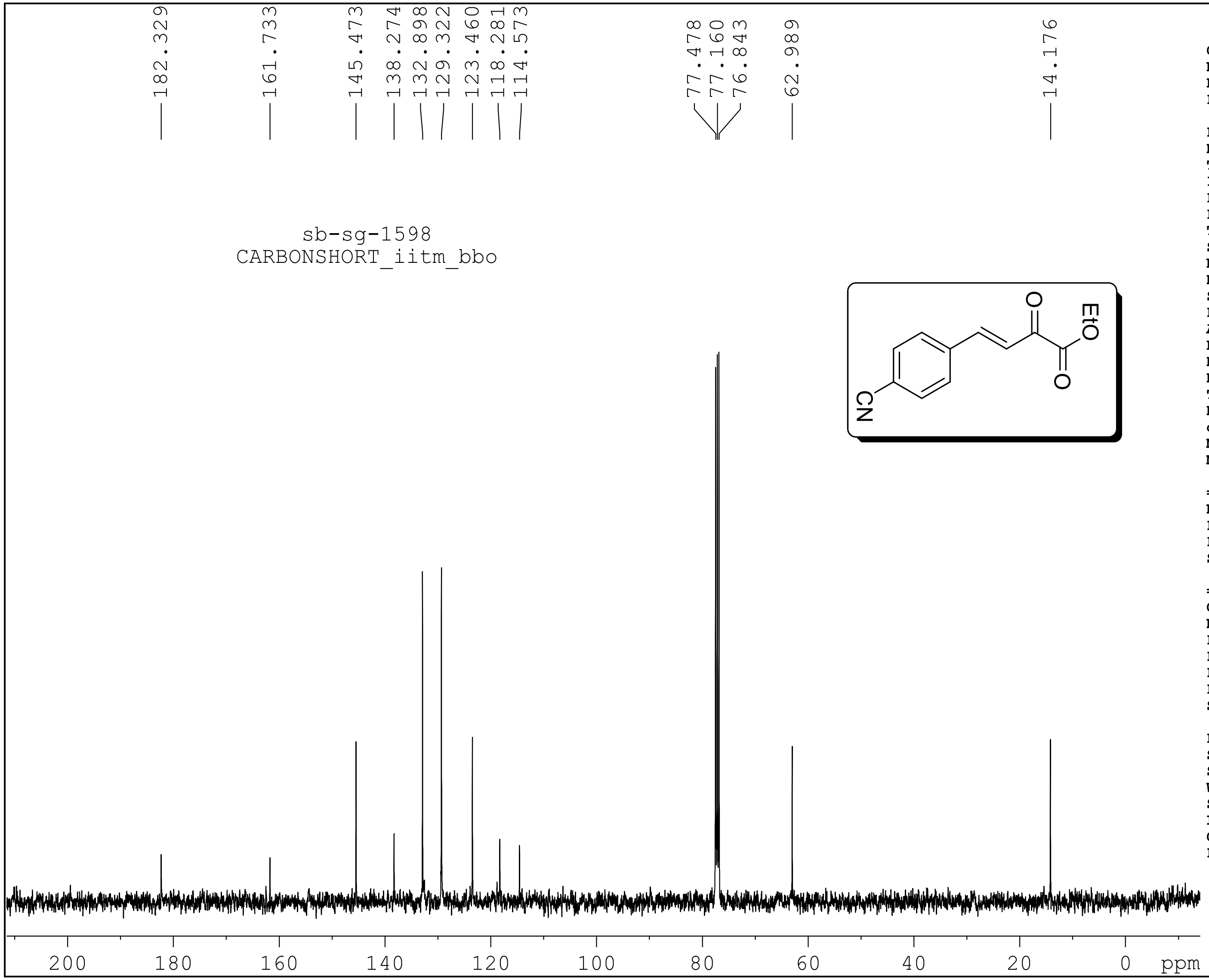


```
Current Data Parameters
NAME              1598
EXPNO              266
PROCNO               1

F2 - Acquisition Parameters
Date_          20110820
Time              13.10
INSTRUM           av400
PROBHD   5 mm BBO BB-1H
PULPROG         zgpg30
TD                16384
SOLVENT           CDCl3
NS                  304
DS                    4
SWH           25125.629 Hz
FIDRES         1.533547 Hz
AQ            0.3260916 sec
RG                 8192
DW               19.900 usec
DE                 6.00 usec
TE          0 K
D1           1.00000000 sec
d11          0.03000000 sec
MCREST      0 sec
MCWRK        0.01500000 sec

======== CHANNEL f1 ========
NUC1                13C
P1                12.50 usec
PL1         0 dB
SFO1        100.6238364 MHz

======== CHANNEL f2 ========
CPDPRG2         waltz16
NUC2                 1H
PCPD2             80.00 usec
PL2                3.00 dB
PL12              19.81 dB
PL13              20.23 dB
SFO2        400.1320007 MHz

F2 - Processing parameters
SI                65536
SF          100.6127599 MHz
WDW                  EM
SSB         0
LB                 4.00 Hz
GB          0
PC                 1.40
```

^{13}C NMR spectrum of compound **9**

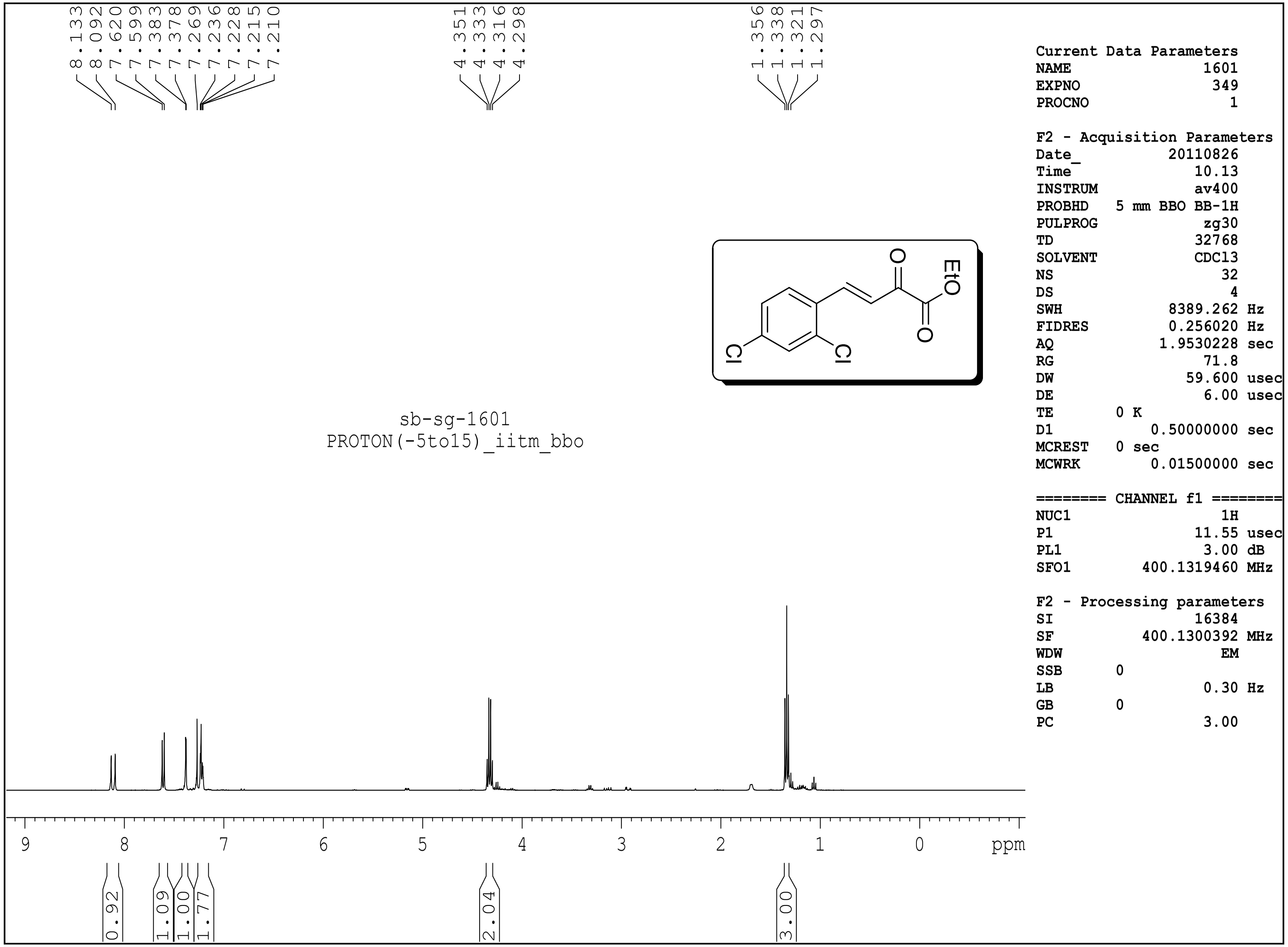

^{1}H NMR spectrum of compound **27**

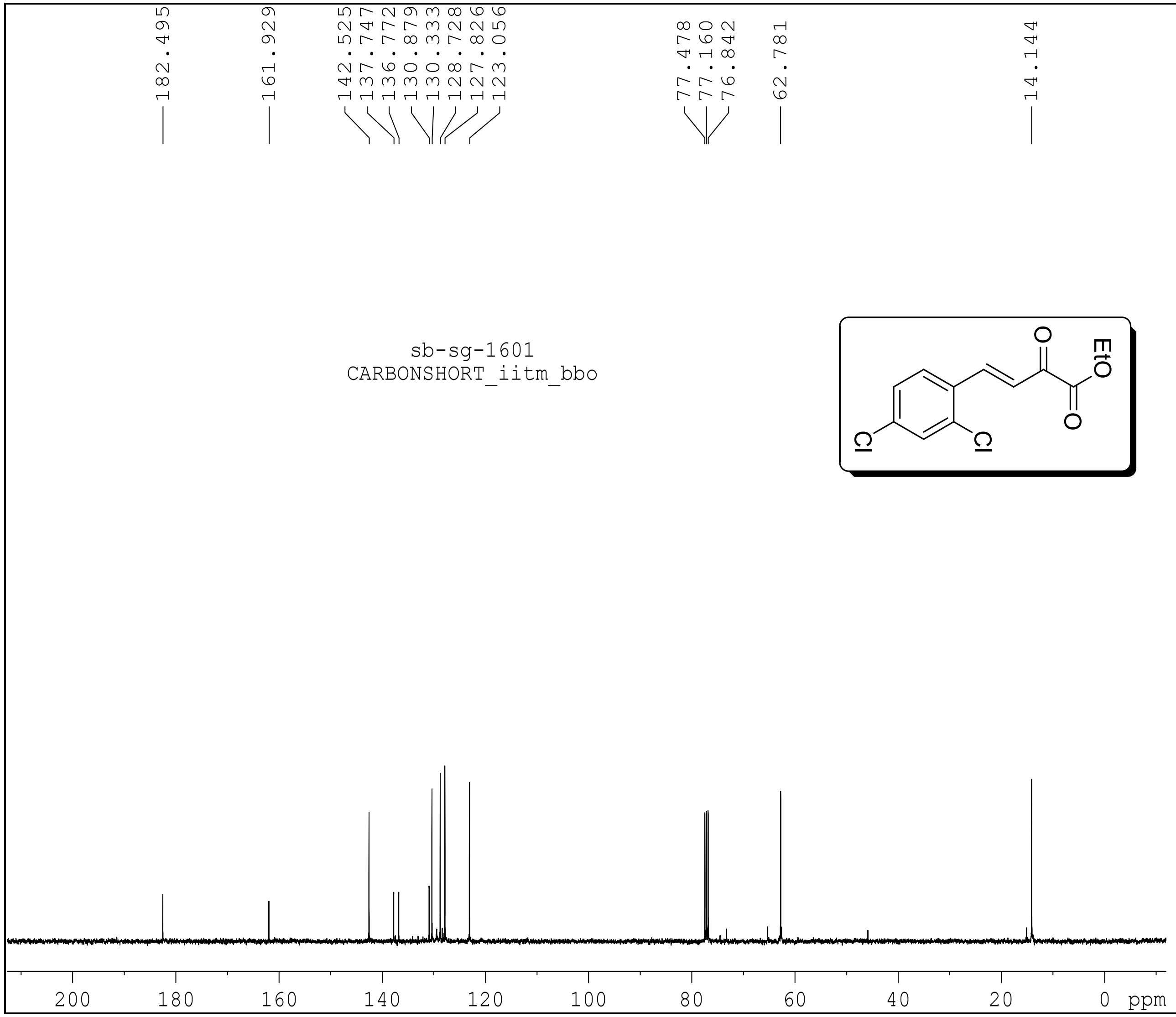


```
Current Data Parameters
NAME                 1601
EXPNO                 350
PROCNO                  1

F2 - Acquisition Parameters
Date_            20110826
Time                10.19
INSTRUM             av400
PROBHD   5 mm BBO BB-1H
PULPROG           zgpg30
TD                 16384
SOLVENT            CDCl3
NS                   303
DS                     4
SWH            25125.629 Hz
FIDRES          1.533547 Hz
AQ             0.3260916 sec
RG                 13004
DW                19.900 usec
DE                  6.00 usec
TE          0 K
D1            1.00000000 sec
d11           0.03000000 sec
MCREST      0 sec
MCWRK         0.01500000 sec

======== CHANNEL f1 ========
NUC1                 13C
P1                 12.50 usec
PL1         0 dB
SFO1         100.6238364 MHz

======== CHANNEL f2 ========
CPDPRG2          waltz16
NUC2                  1H
PCPD2              80.00 usec
PL2                 3.00 dB
PL12               19.81 dB
PL13               20.23 dB
SFO2         400.1320007 MHz

F2 - Processing parameters
SI                 65536
SF           100.6127637 MHz
WDW                   EM
SSB         0
LB                  2.00 Hz
GB          0
PC                  1.40
```

^{13}C NMR spectrum of compound **27**

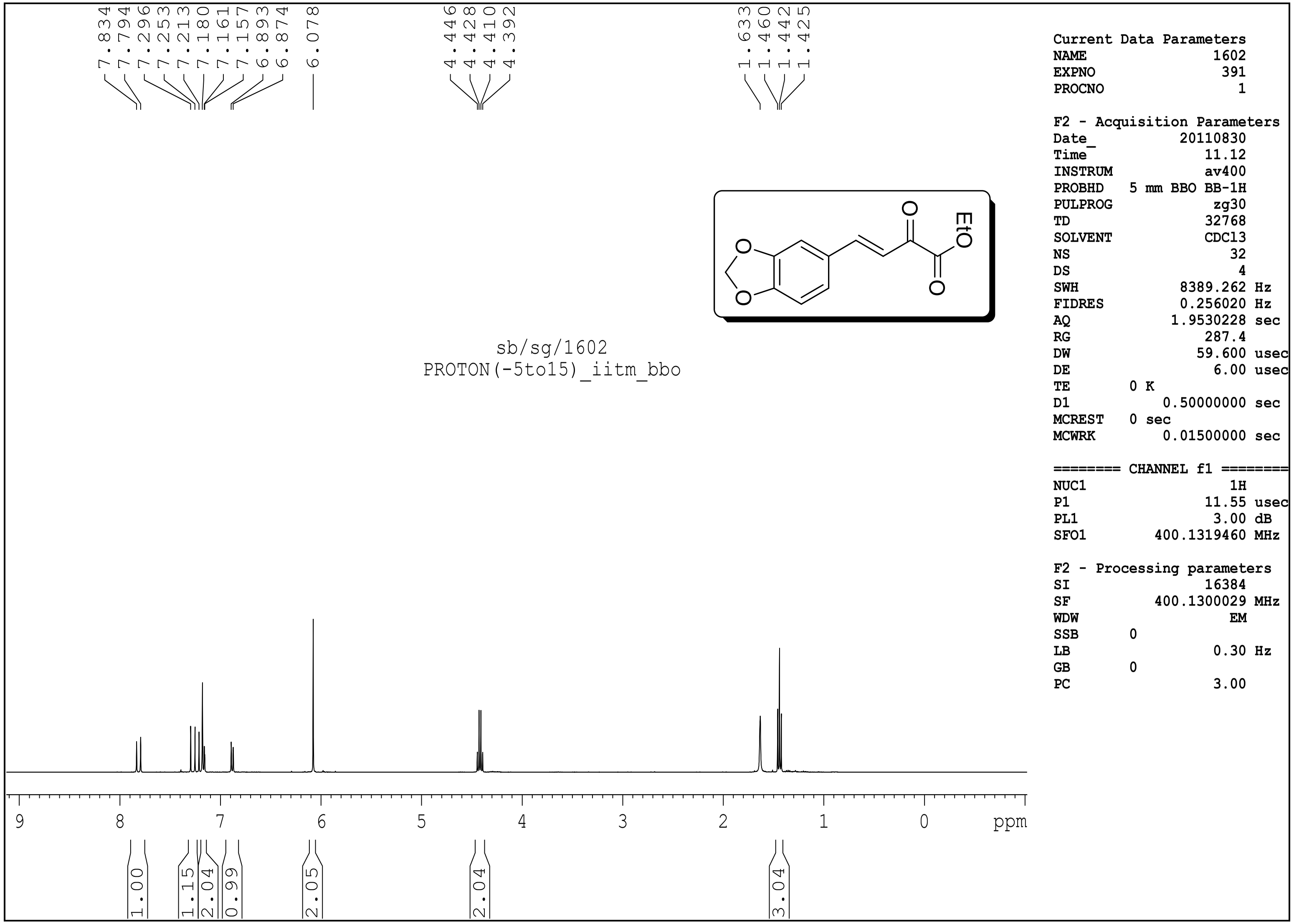

^{1}H NMR spectrum of compound **13**

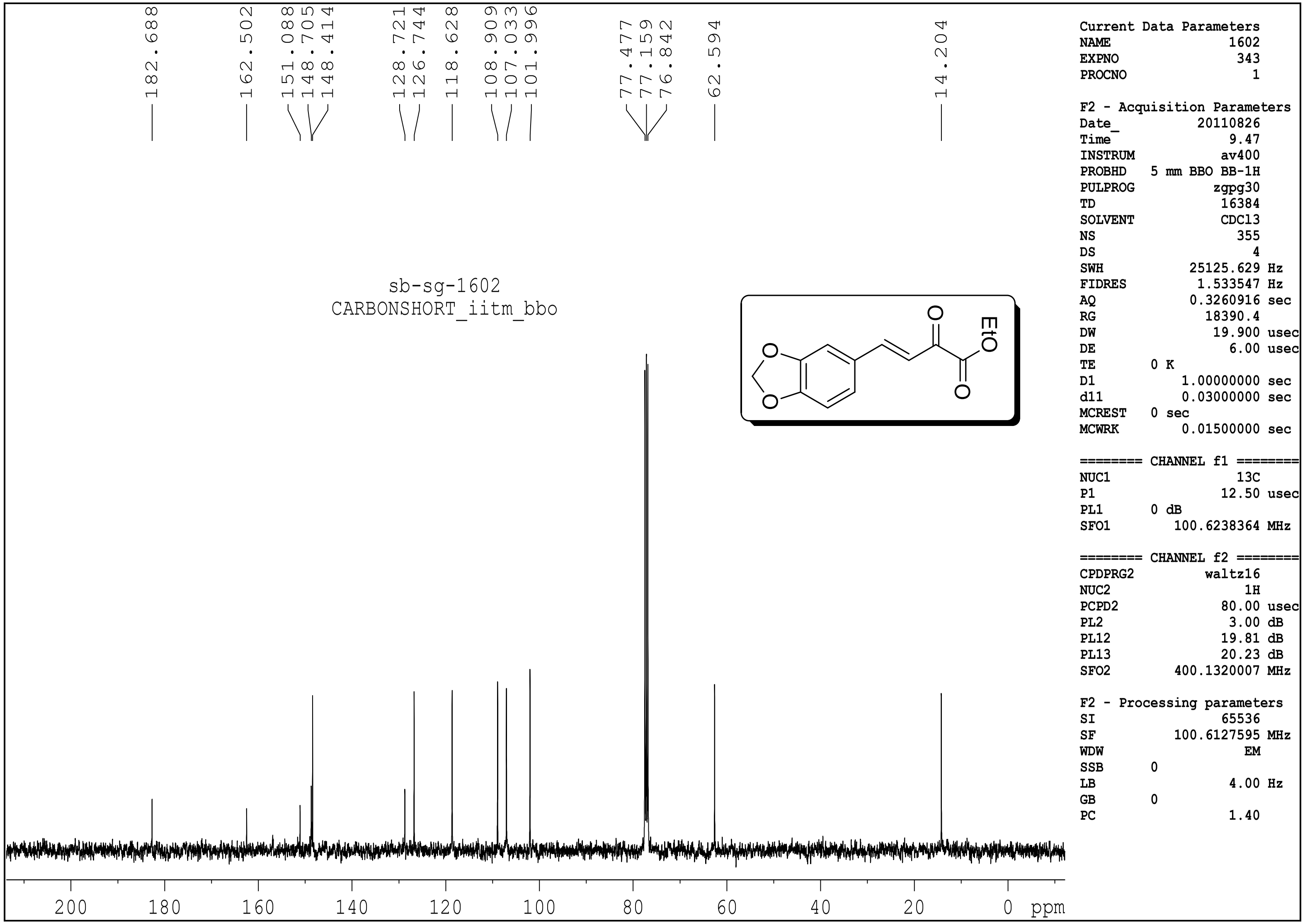

^{13}C NMR spectrum of compound **13**

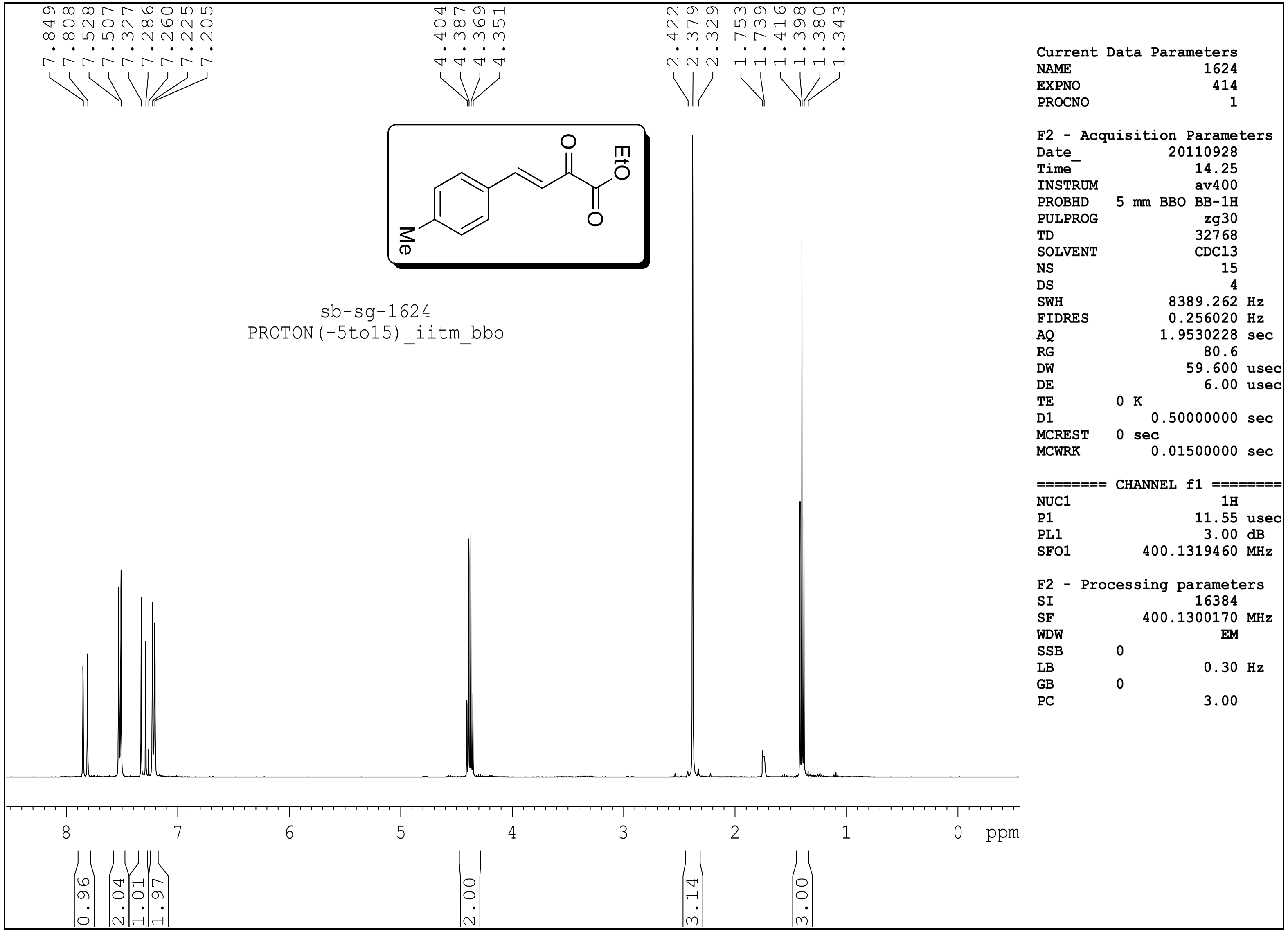

^{1}H NMR spectrum of compound **15**

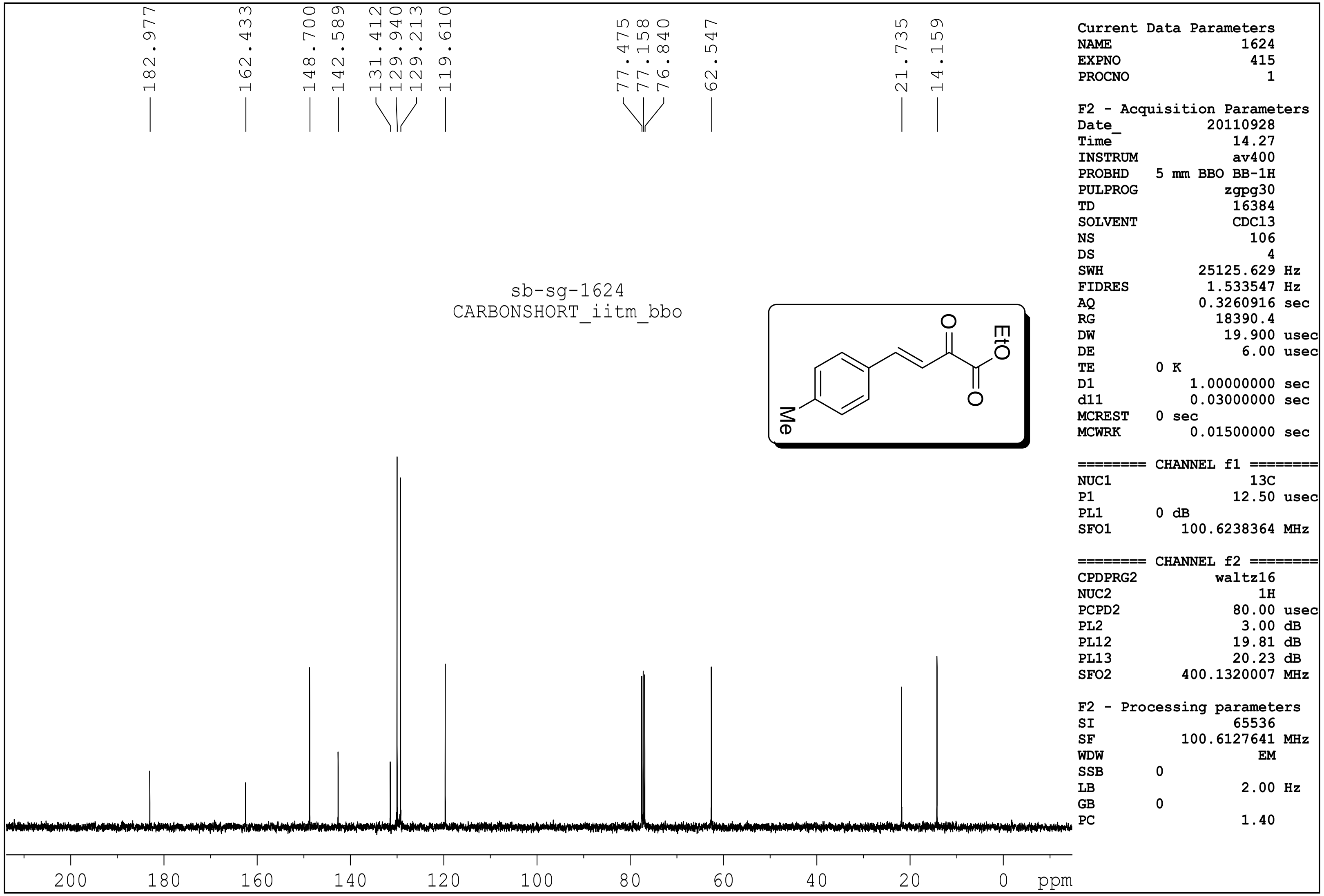

^{13}C NMR spectrum of compound **15**

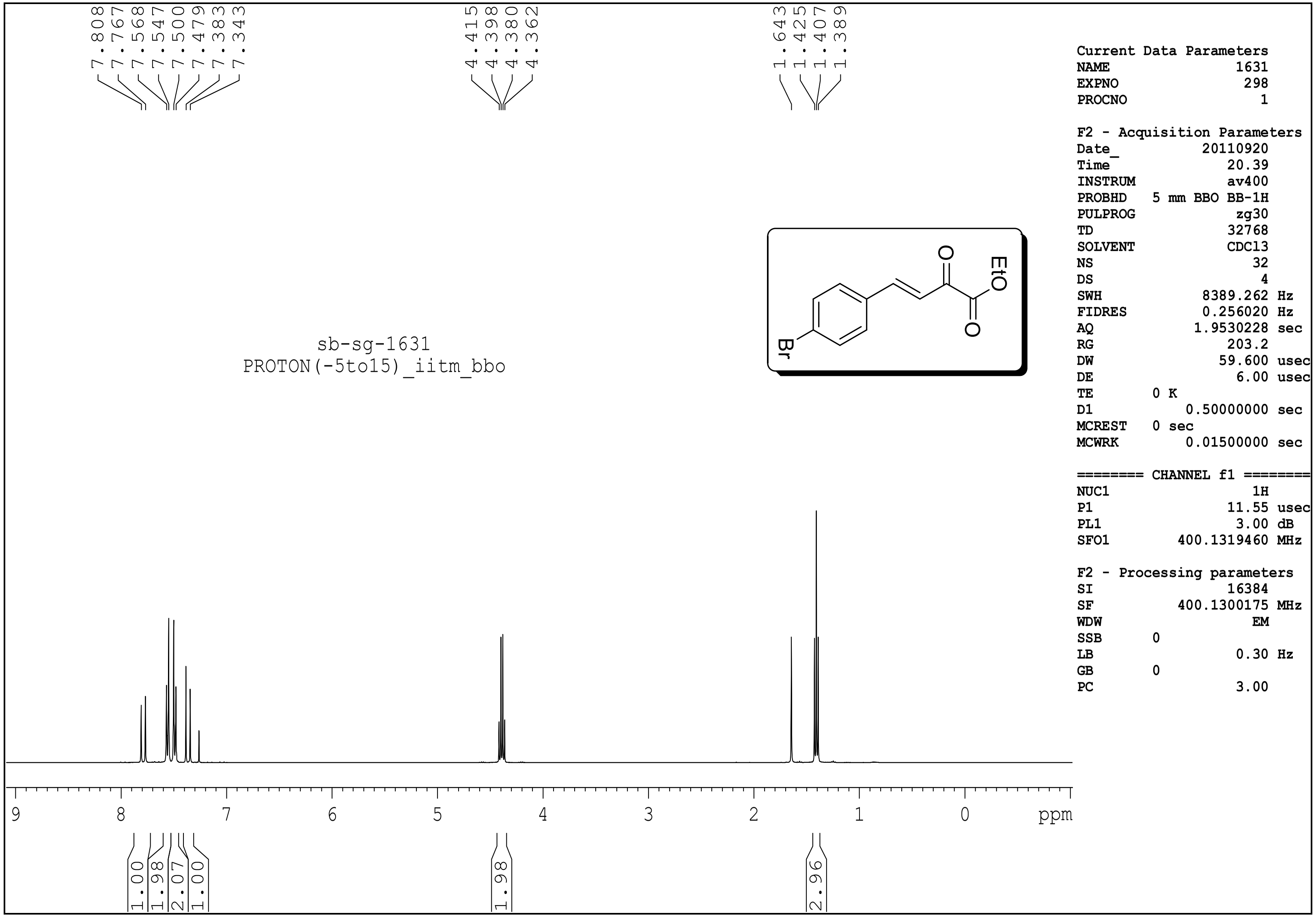

^{1}H NMR spectrum of compound **19**

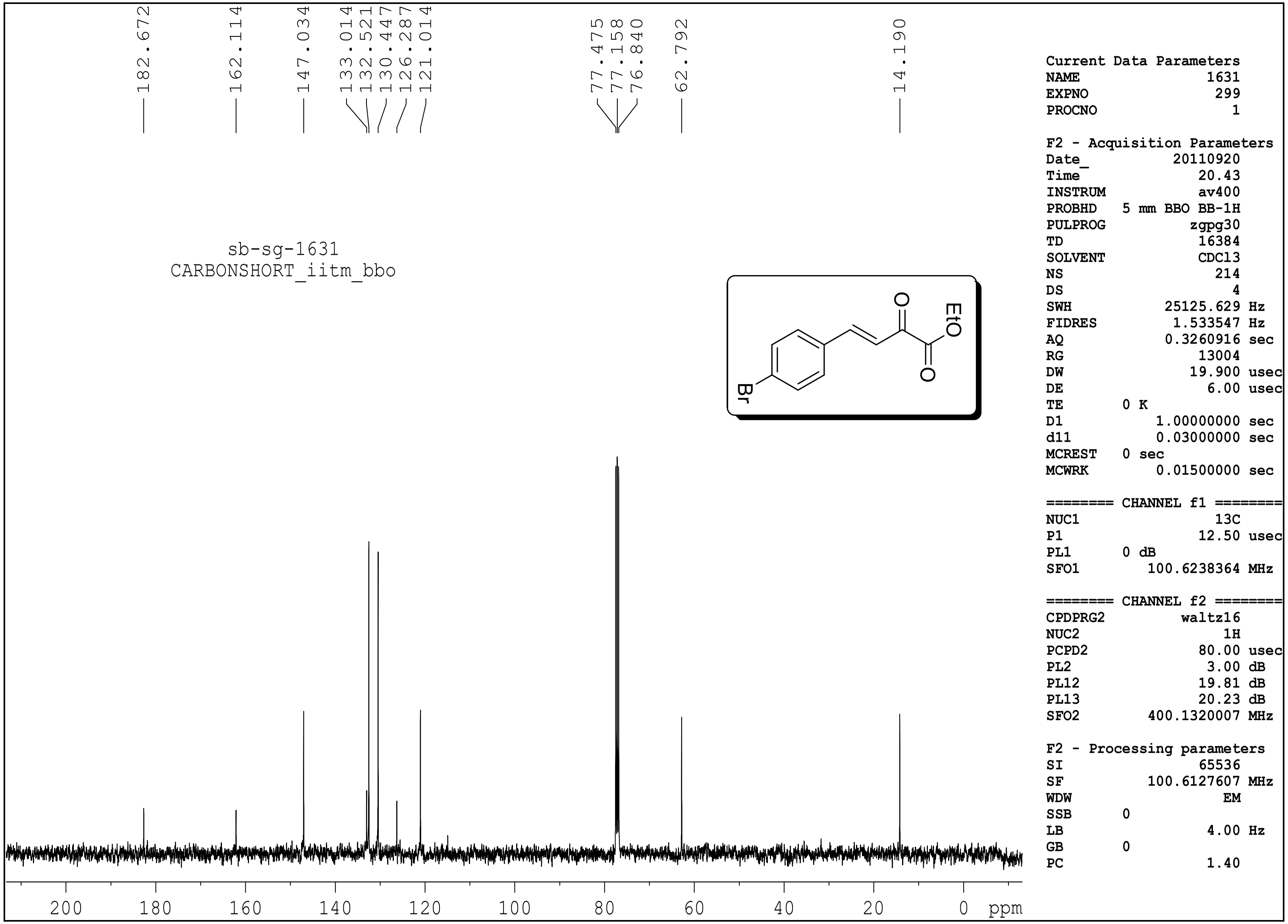

^{13}C NMR spectrum of compound **19**

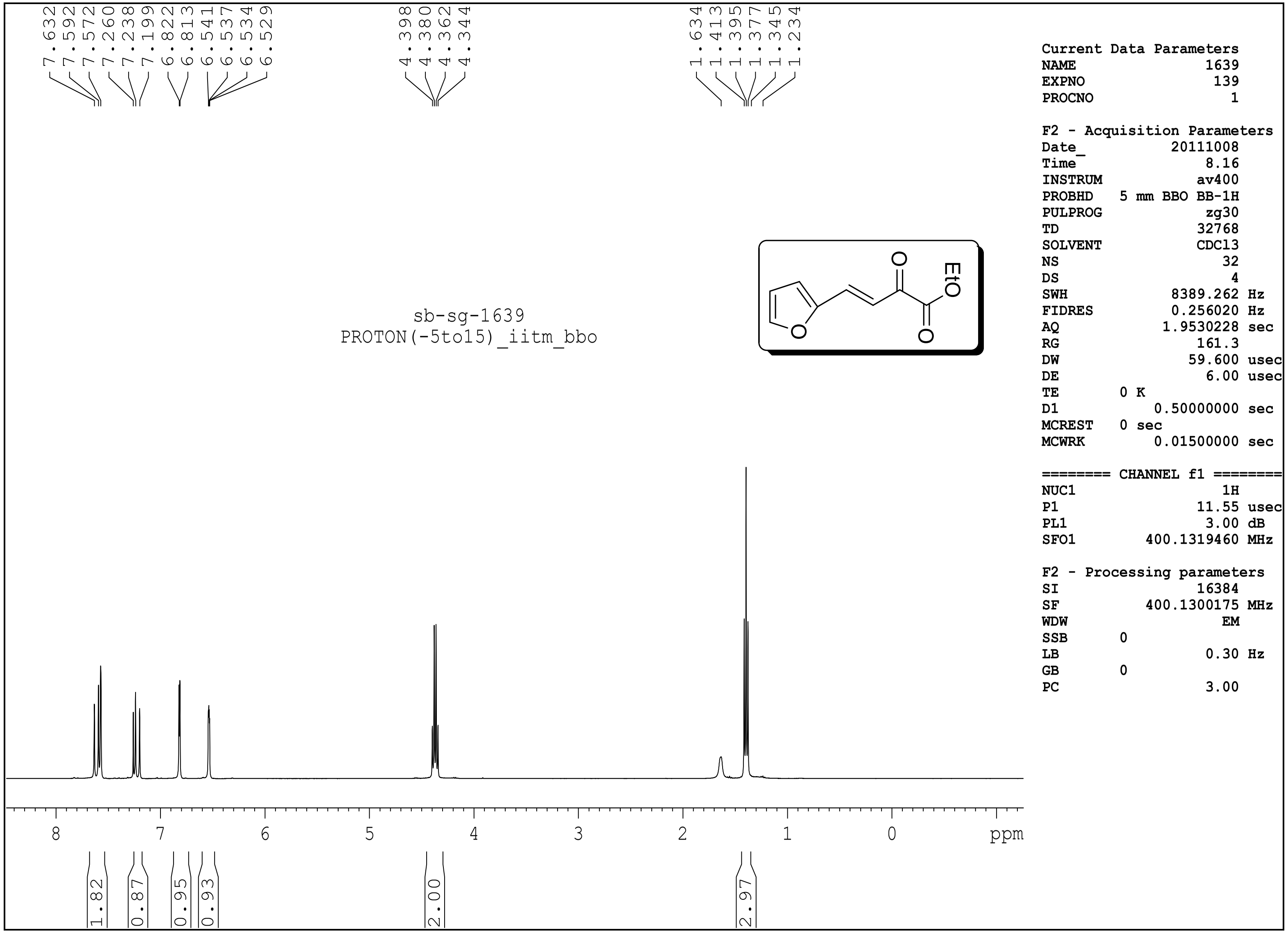

^{1}H NMR spectrum of compound **21**

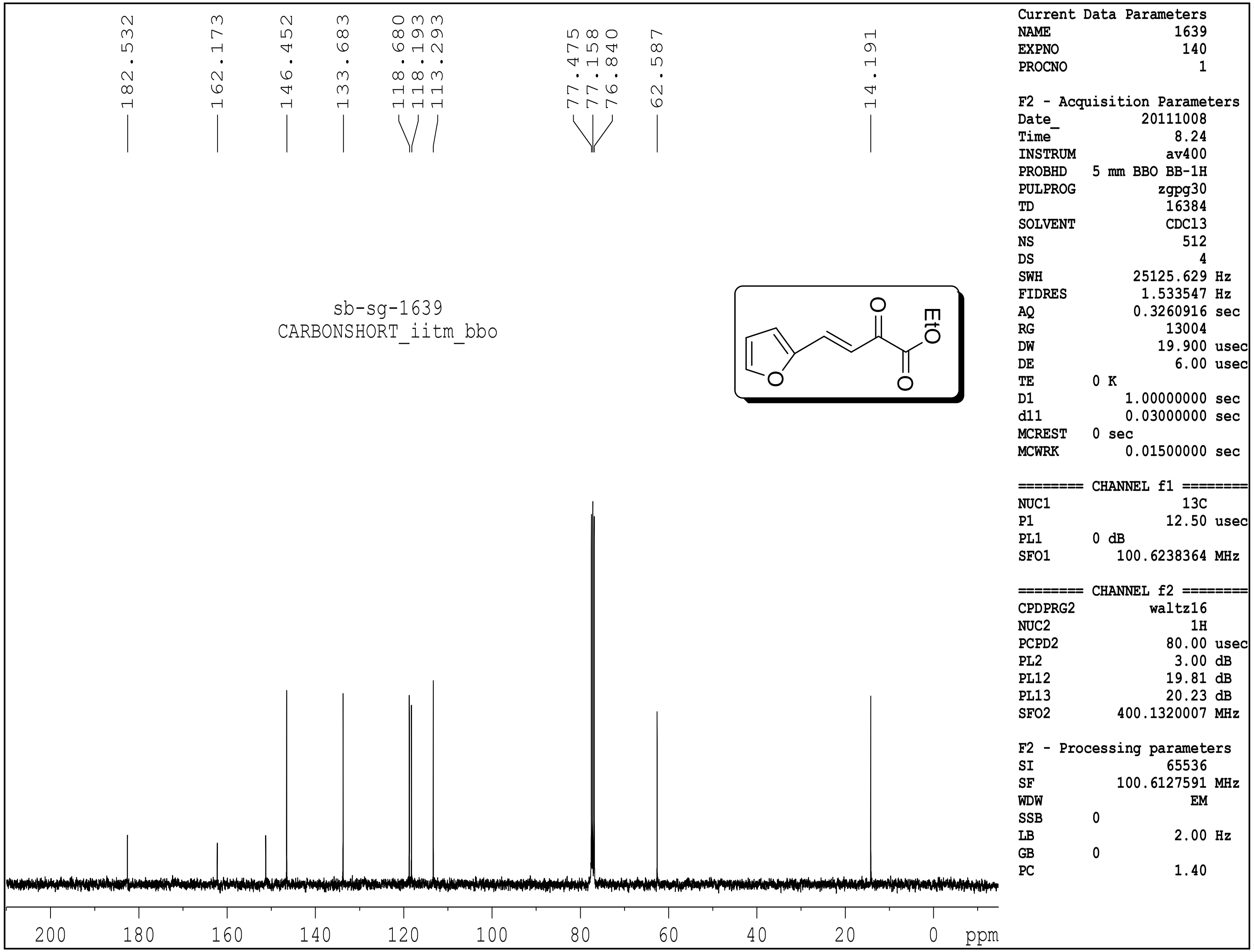

^{13}C NMR spectrum of compound **21**

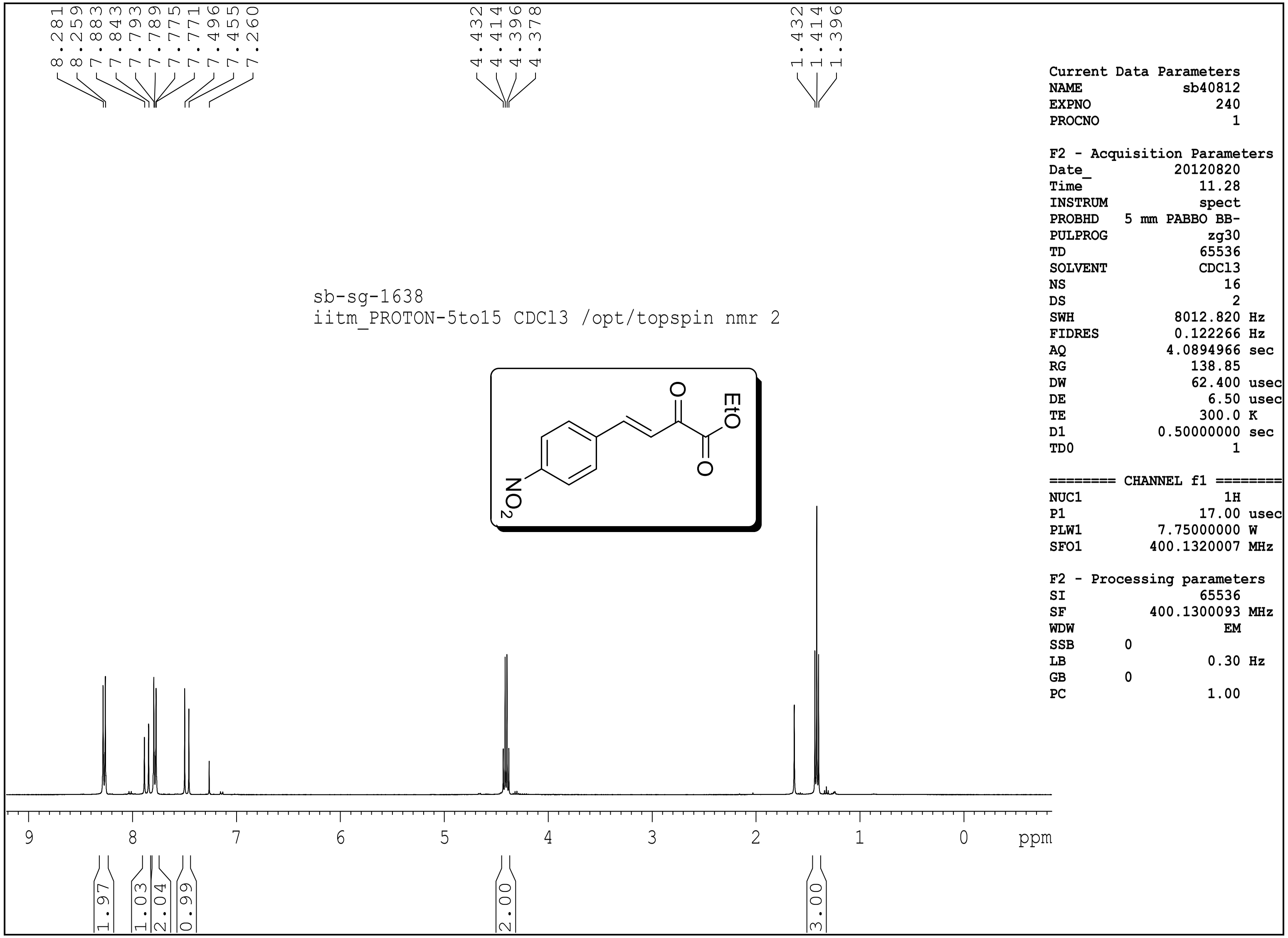

^{1}H NMR spectrum of compound **17**

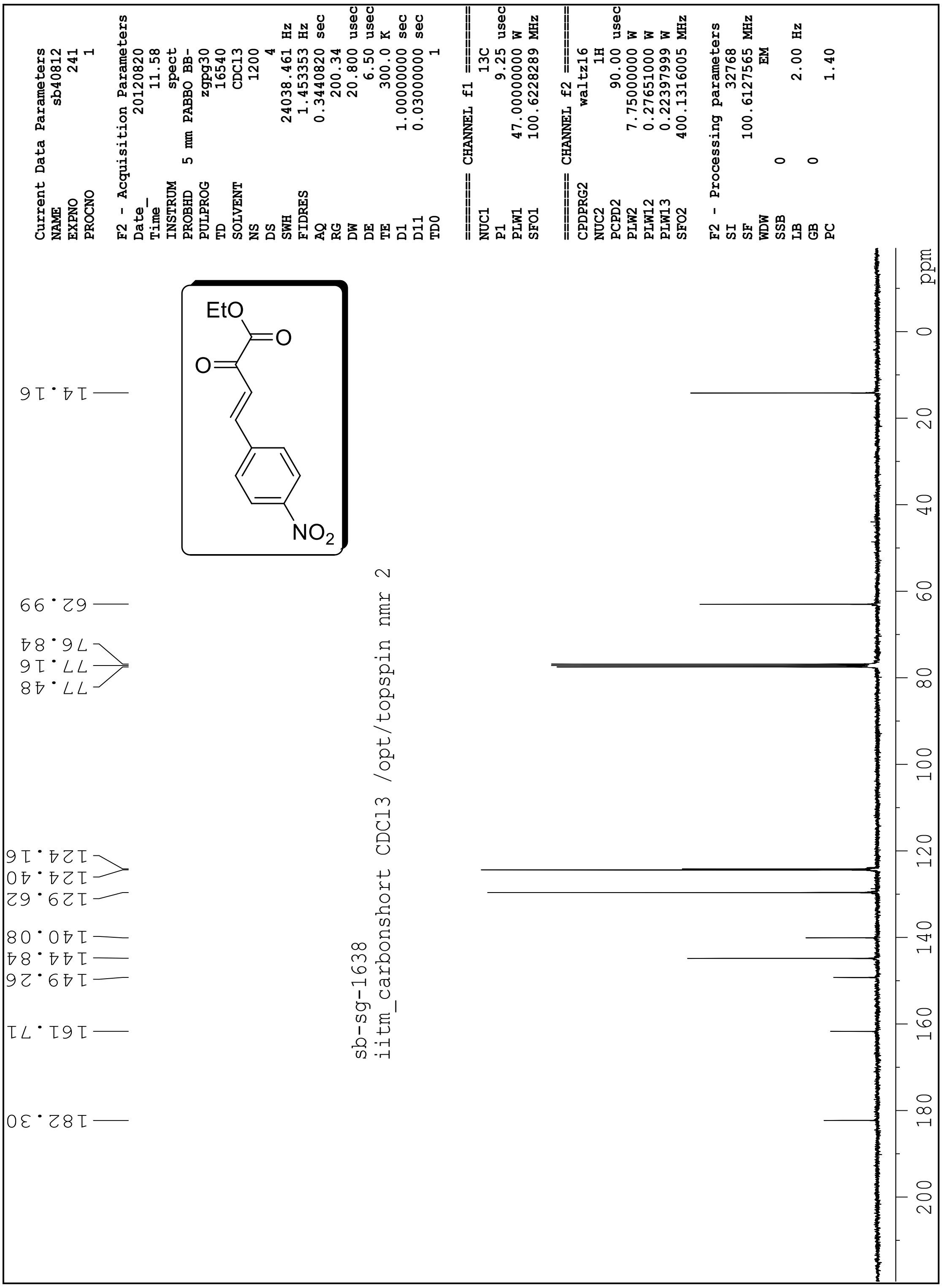

^{13}C NMR spectrum of compound **17**

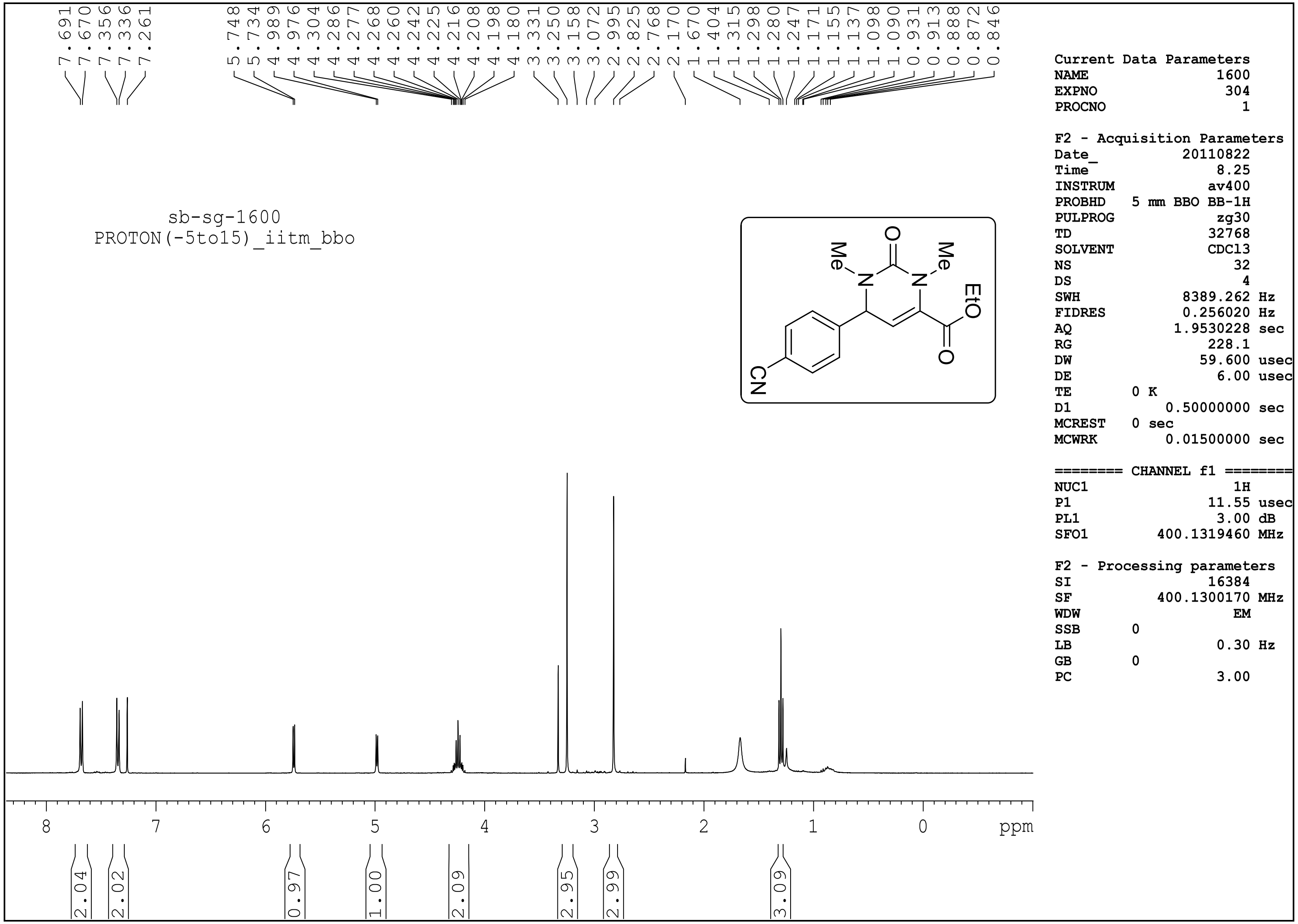

^{1}H NMR spectrum of compound **10**

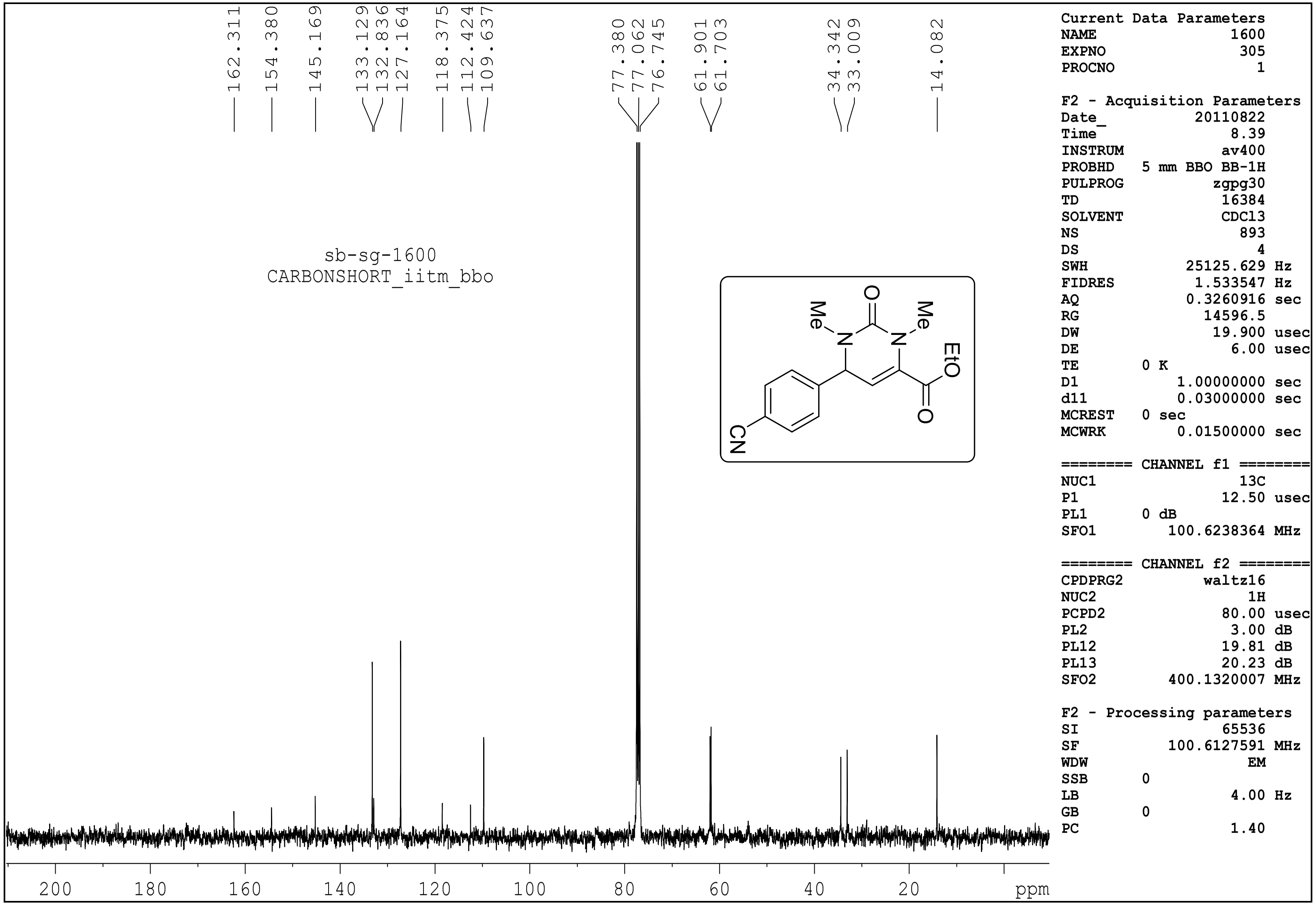

^{13}C NMR spectrum of compound **10**

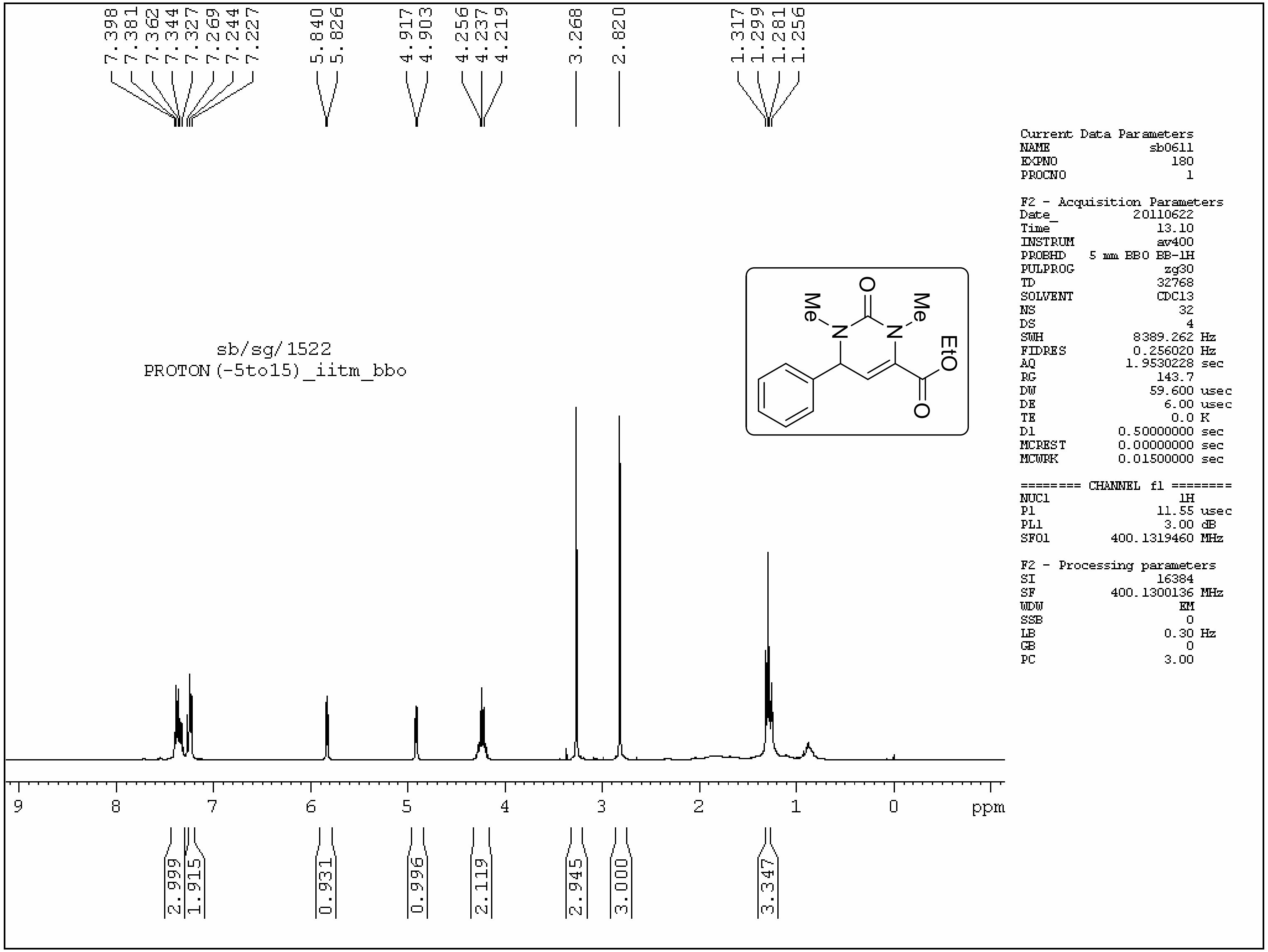

^{1}H NMR spectrum of compound **8**

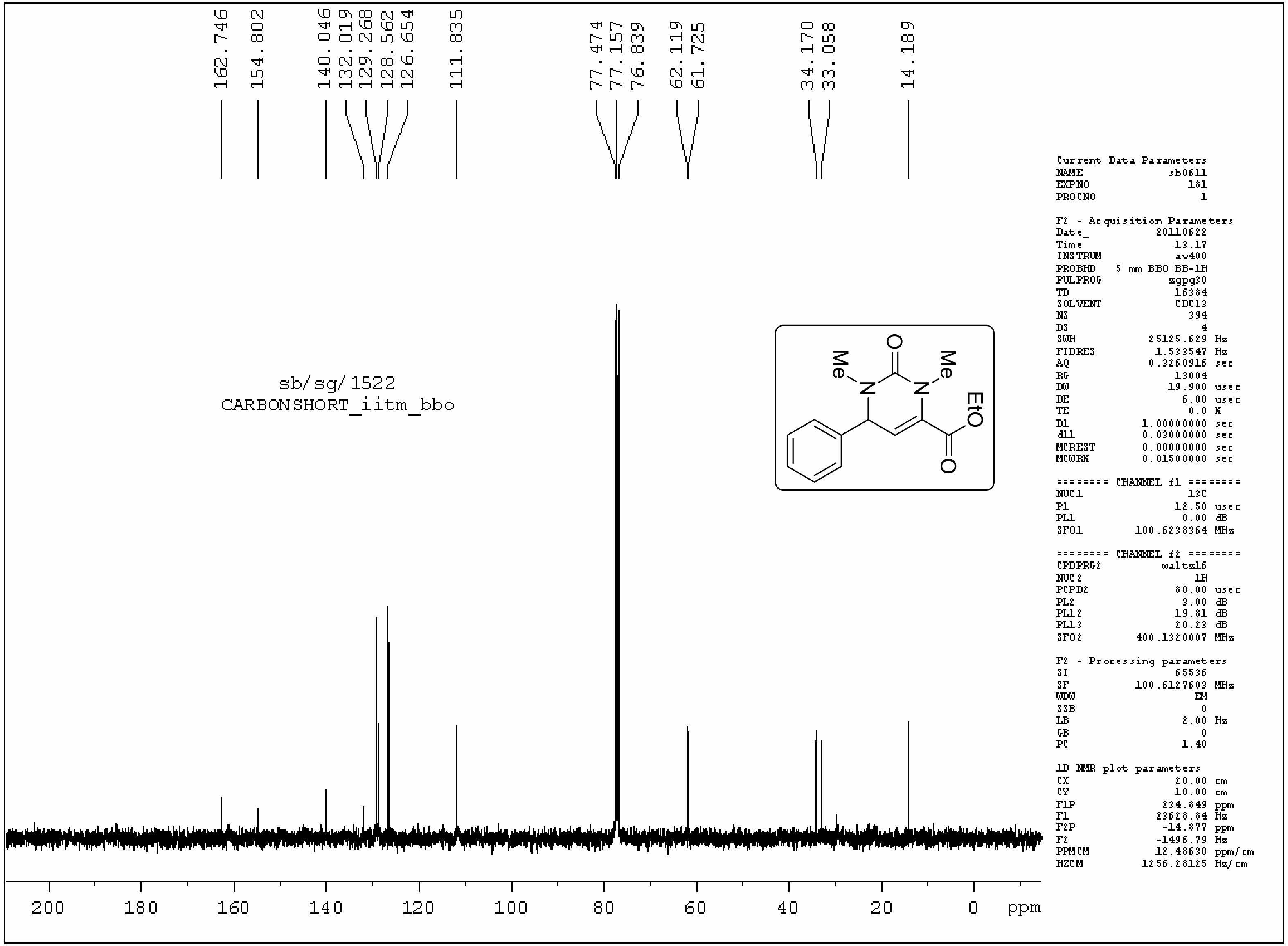

^{13}CNMR spectrum of compound **8**

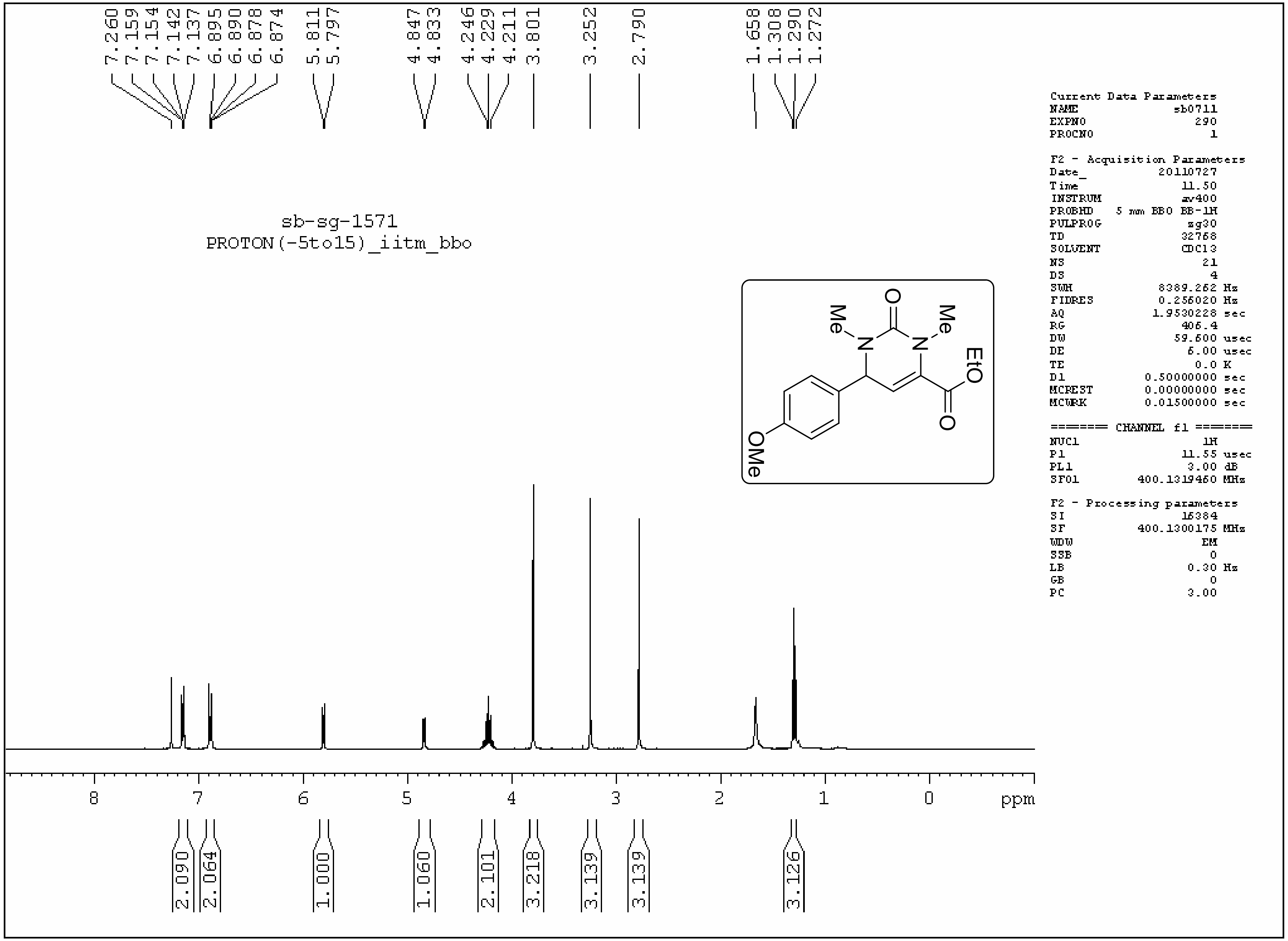

^{1}H NMR spectrum of compound **12**

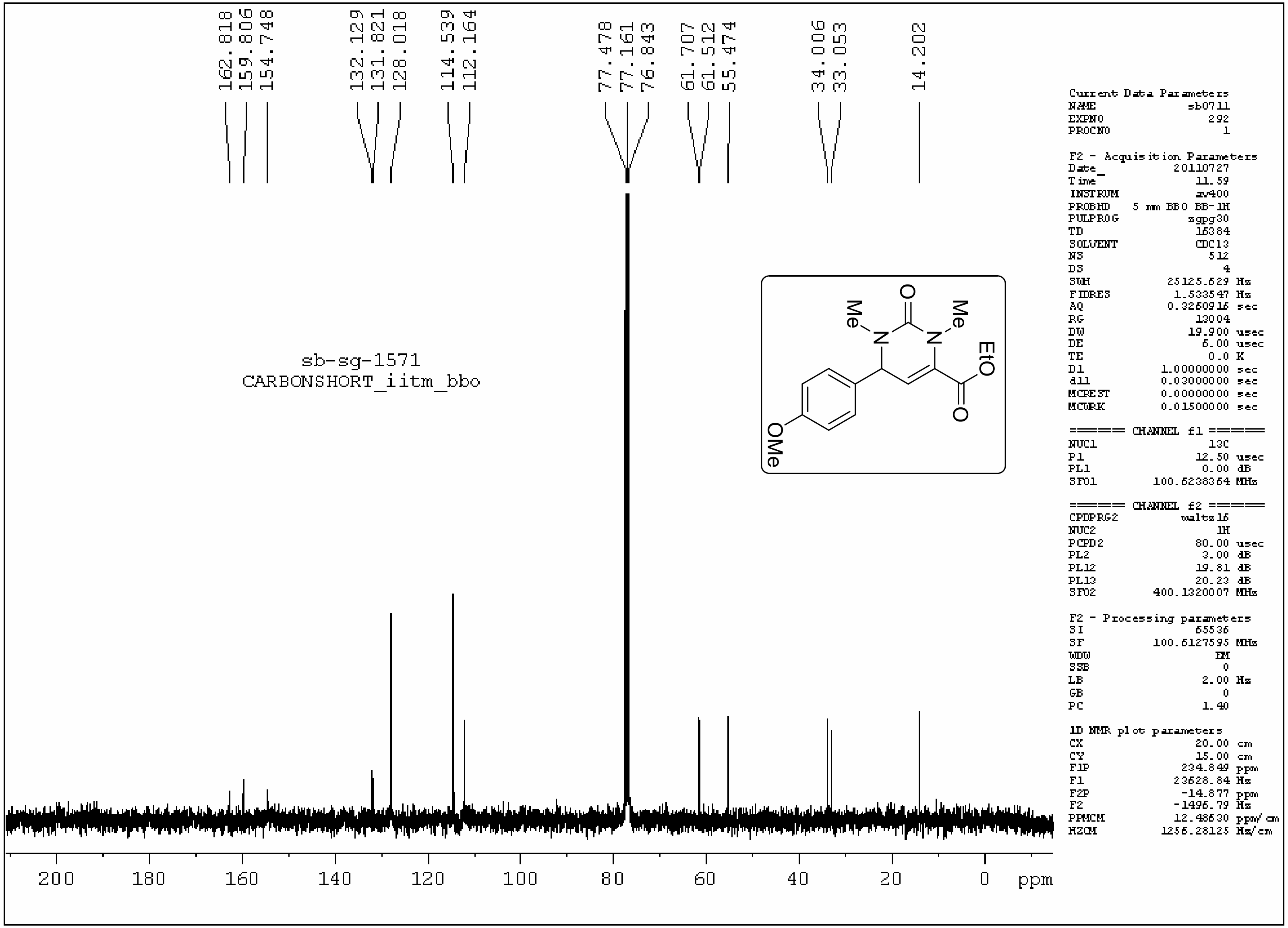

^{13}CNMR spectrum of compound **12**

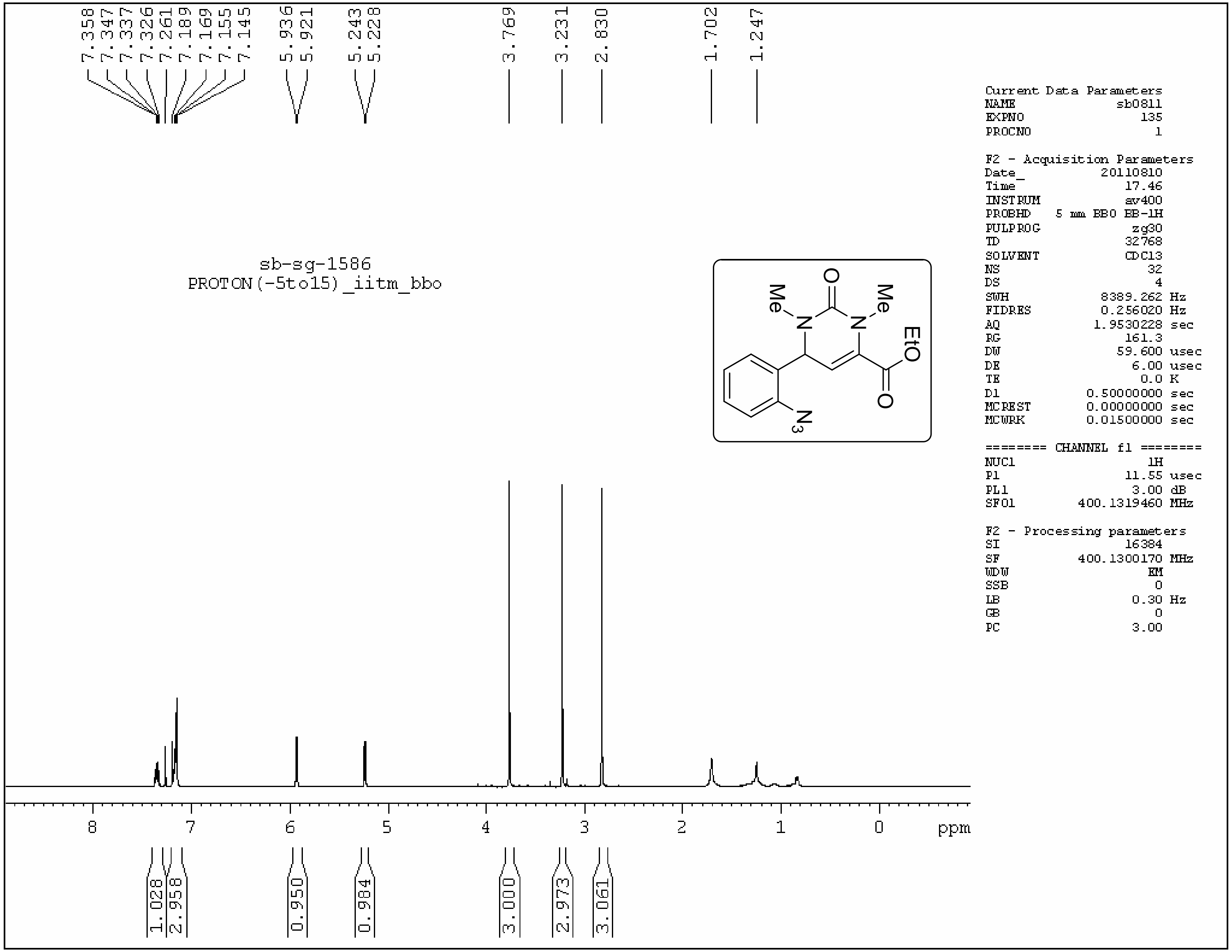

^{1}H NMR spectrum of compound **24**

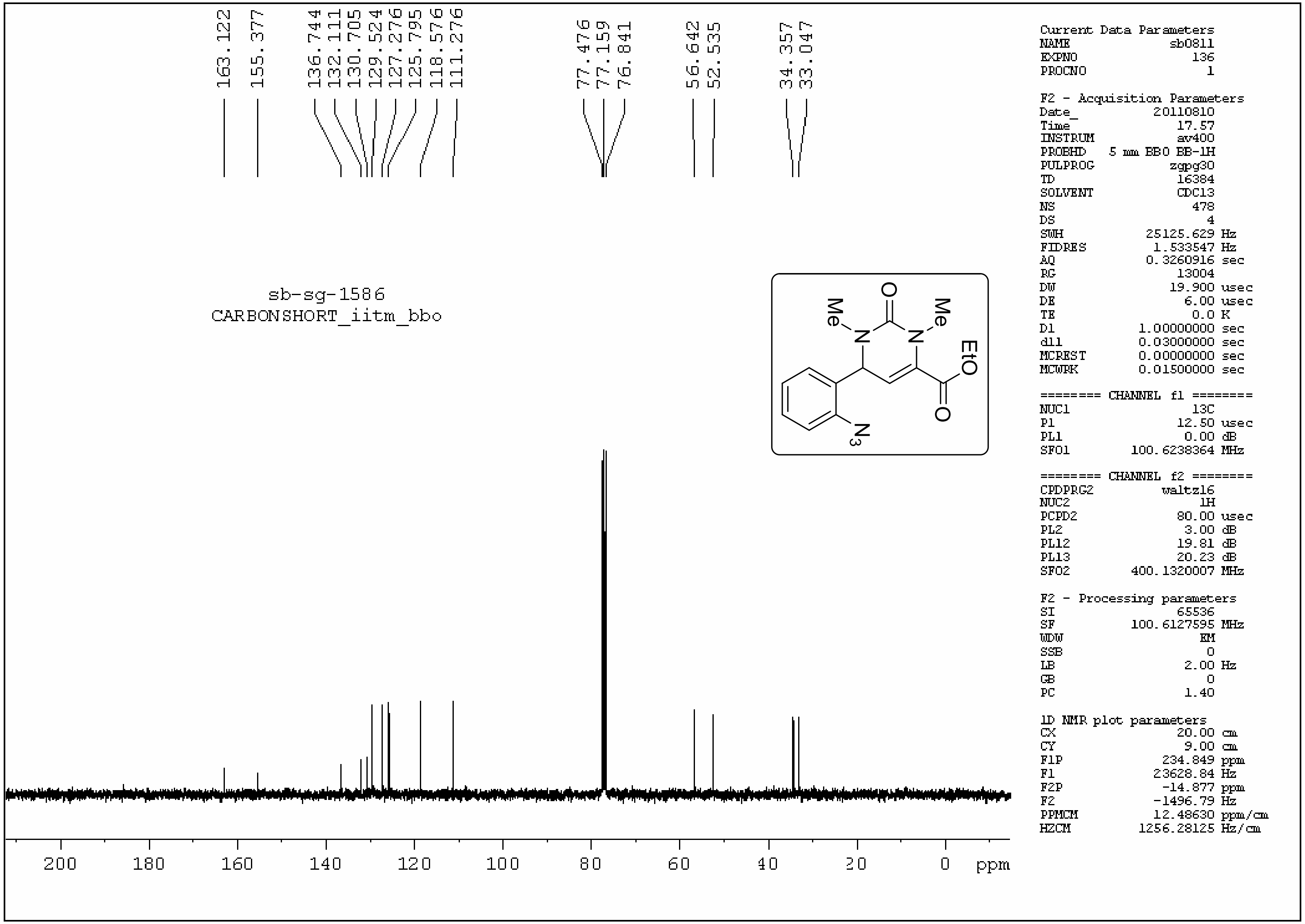

^{13}C NMR spectrum of compound **24**

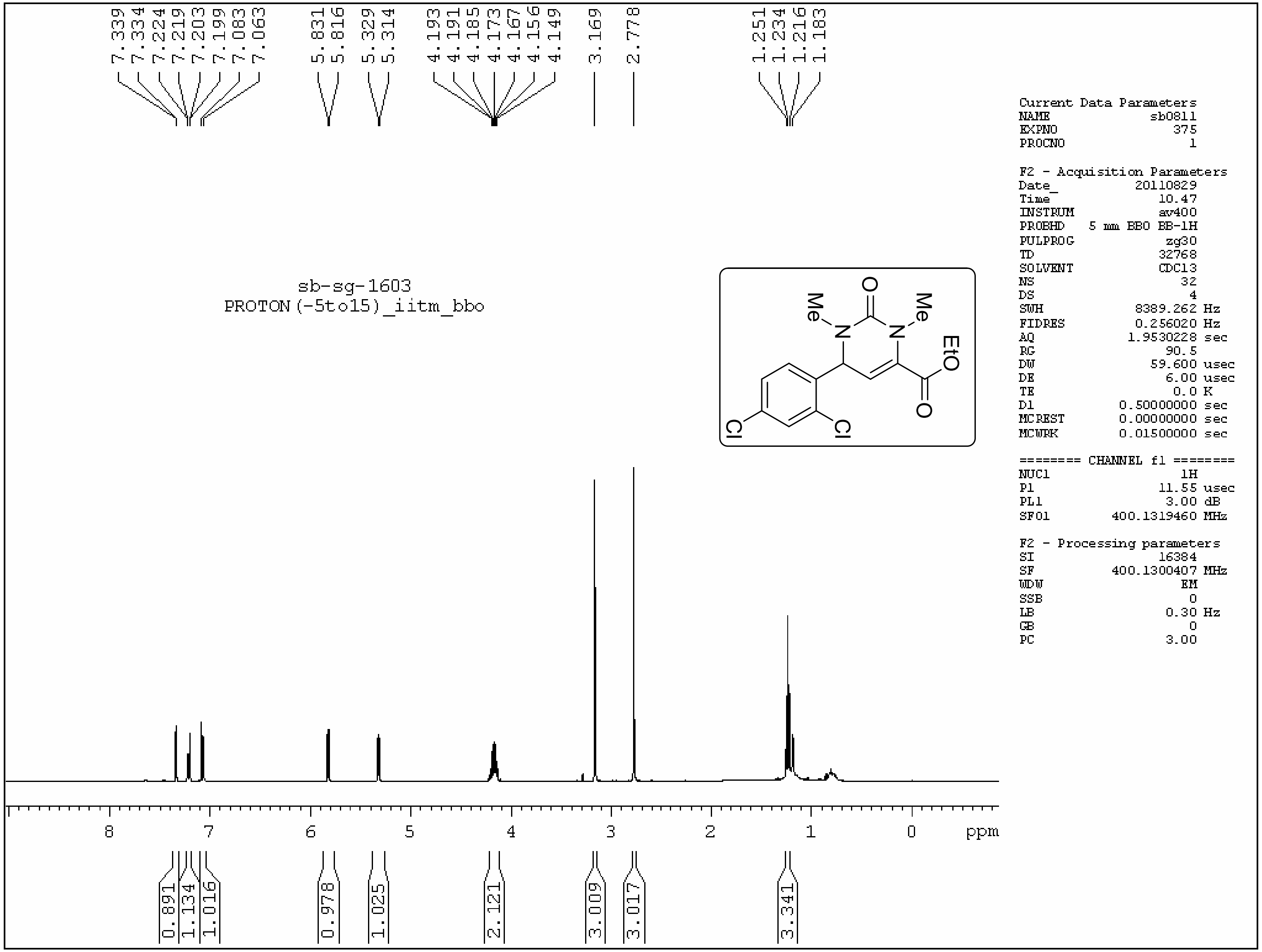

^{1}H NMR spectrum of compound **28**

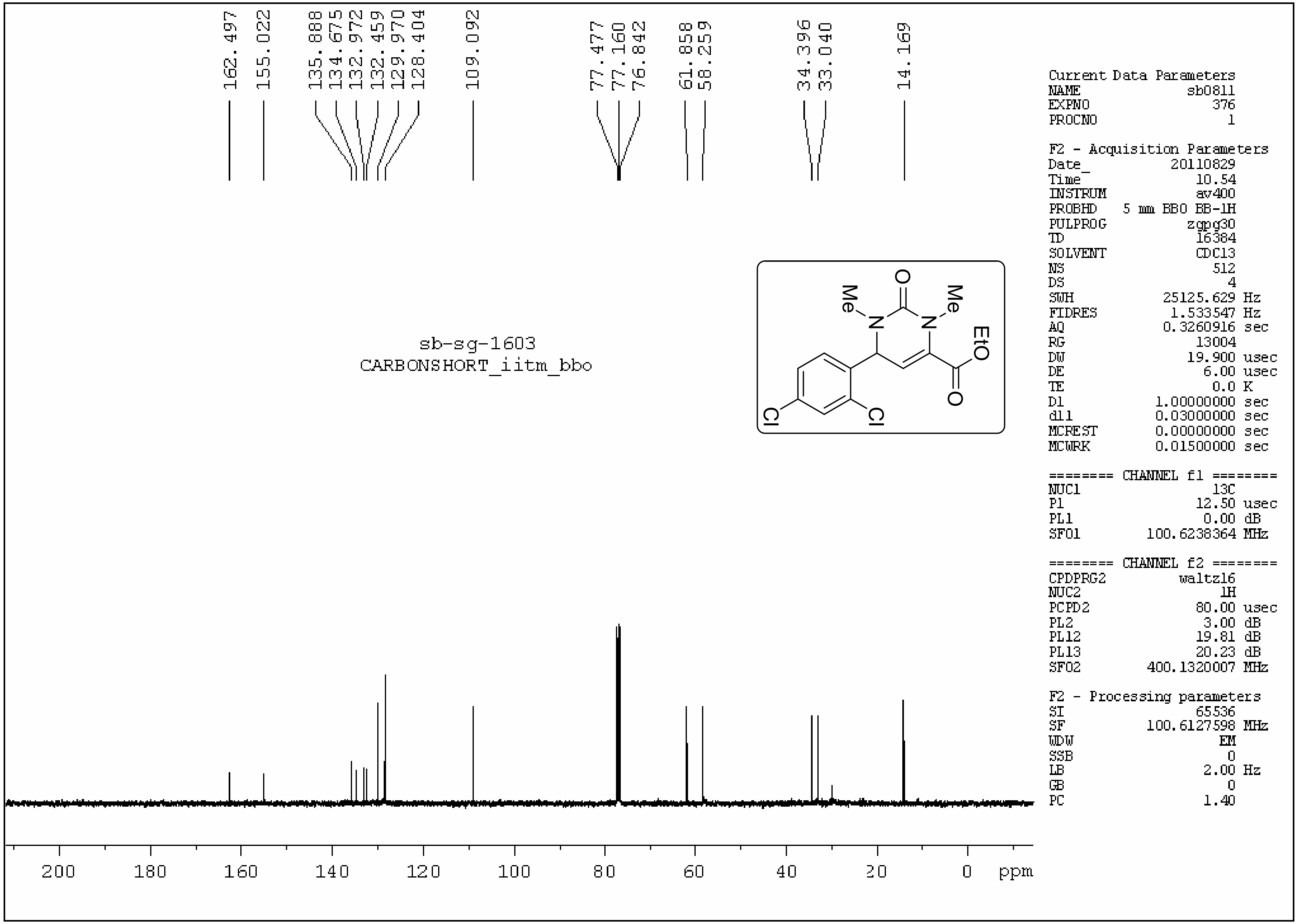

^{13}C NMR spectrum of compound **28**

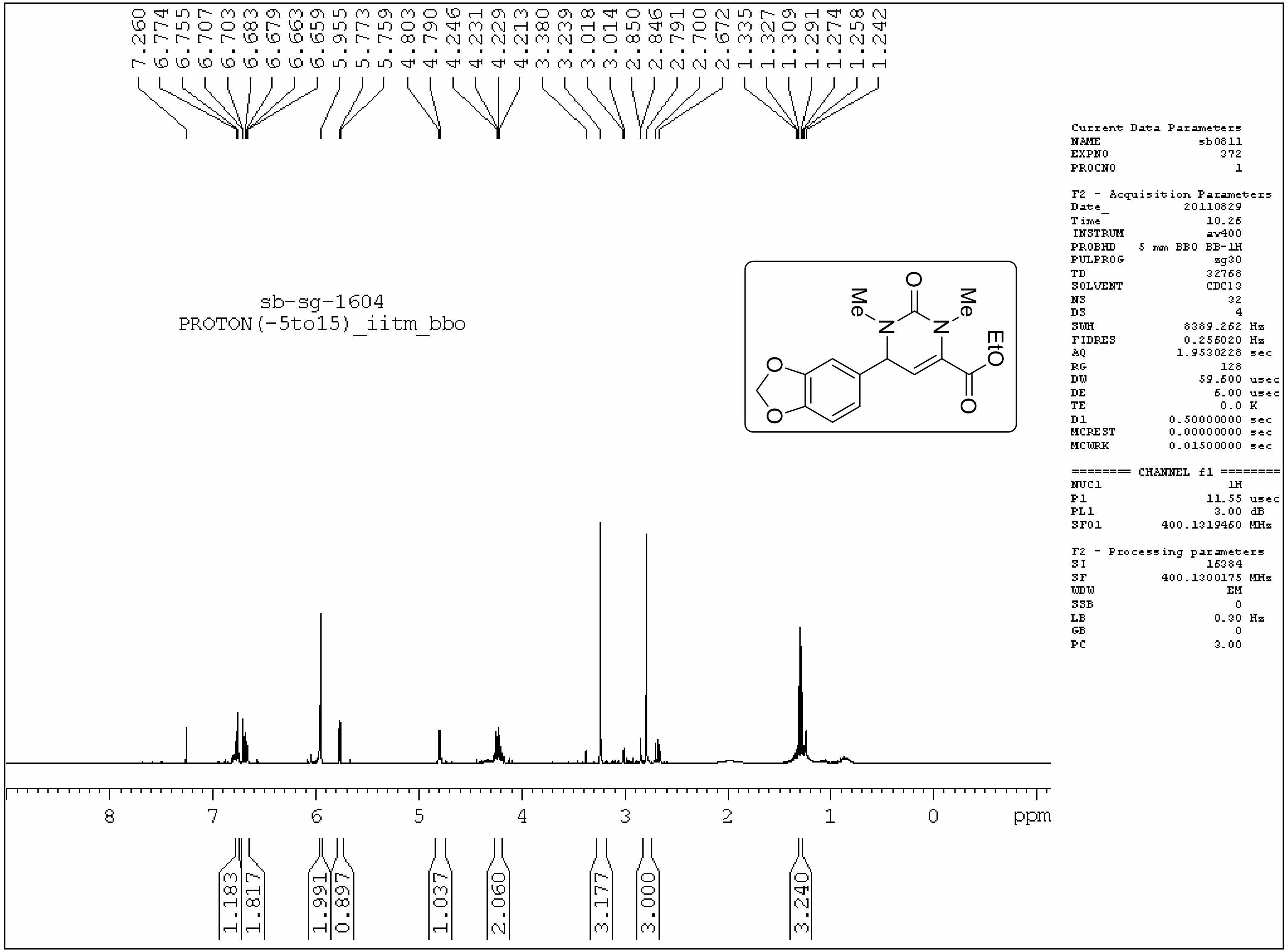

^{1}H NMR spectrum of compound **14**

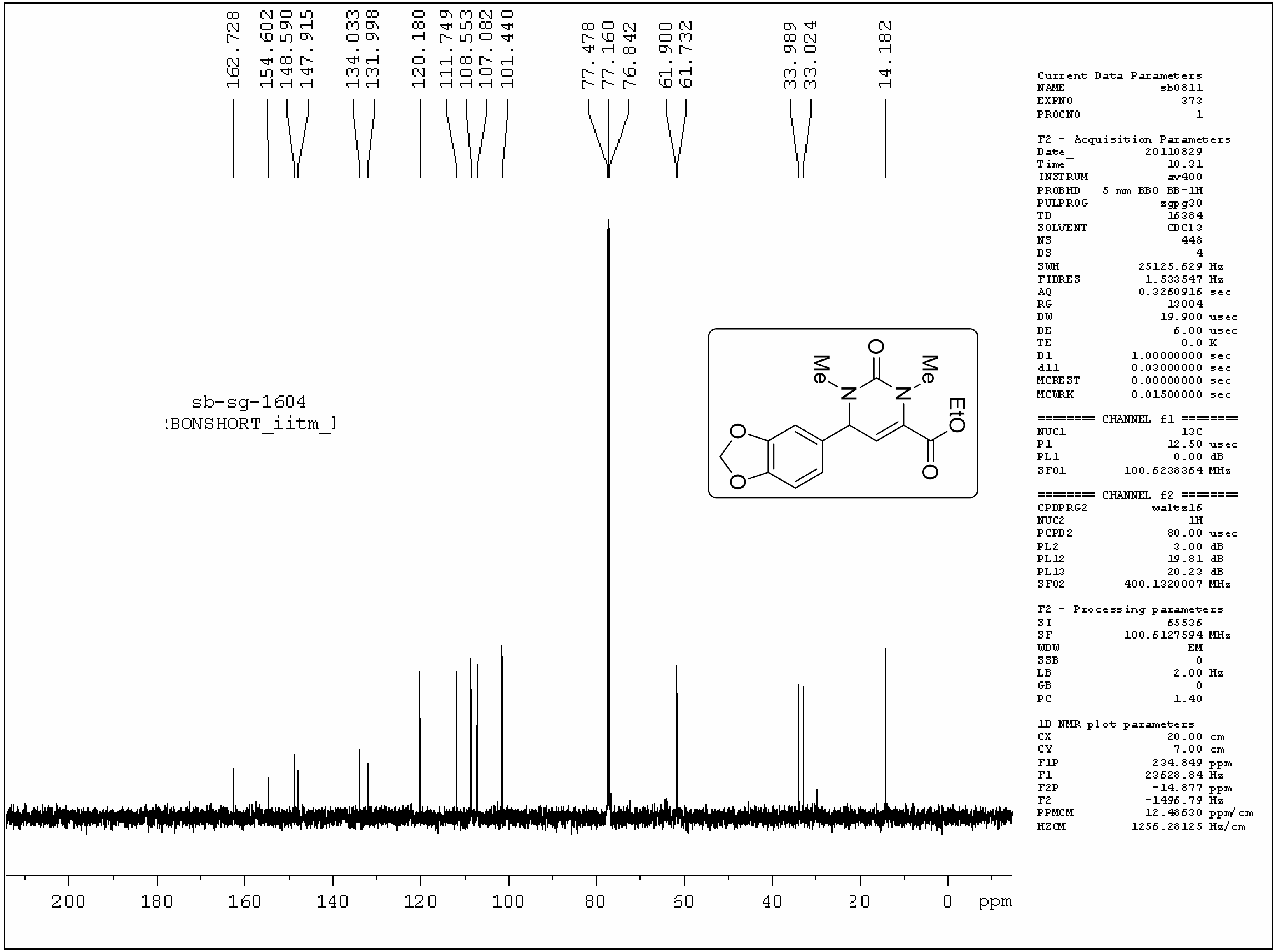

^{13}C NMR spectrum of compound **14**

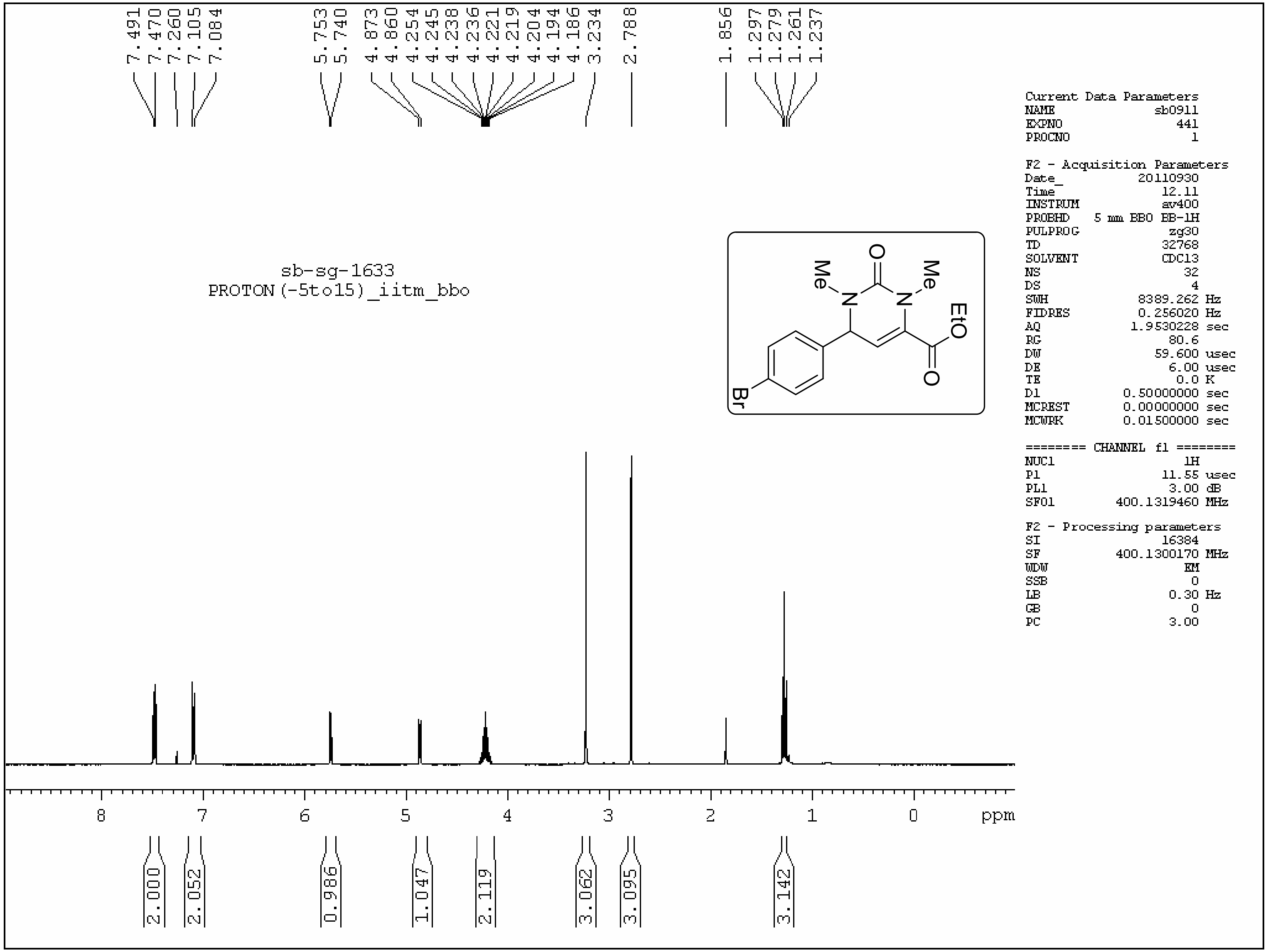

^{1}H NMR spectrum of compound **20**

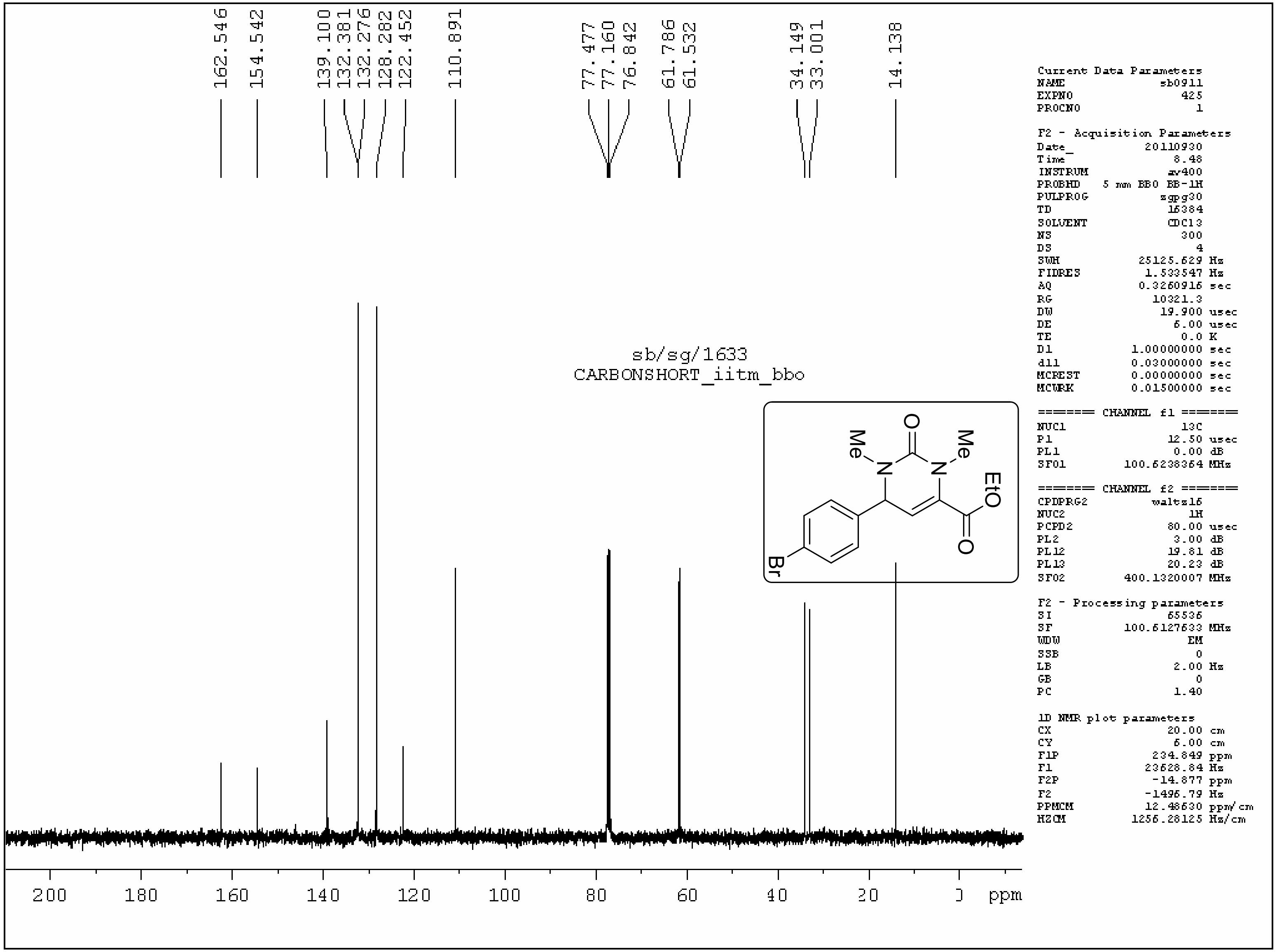

^{13}C NMR spectrum of compound **20**

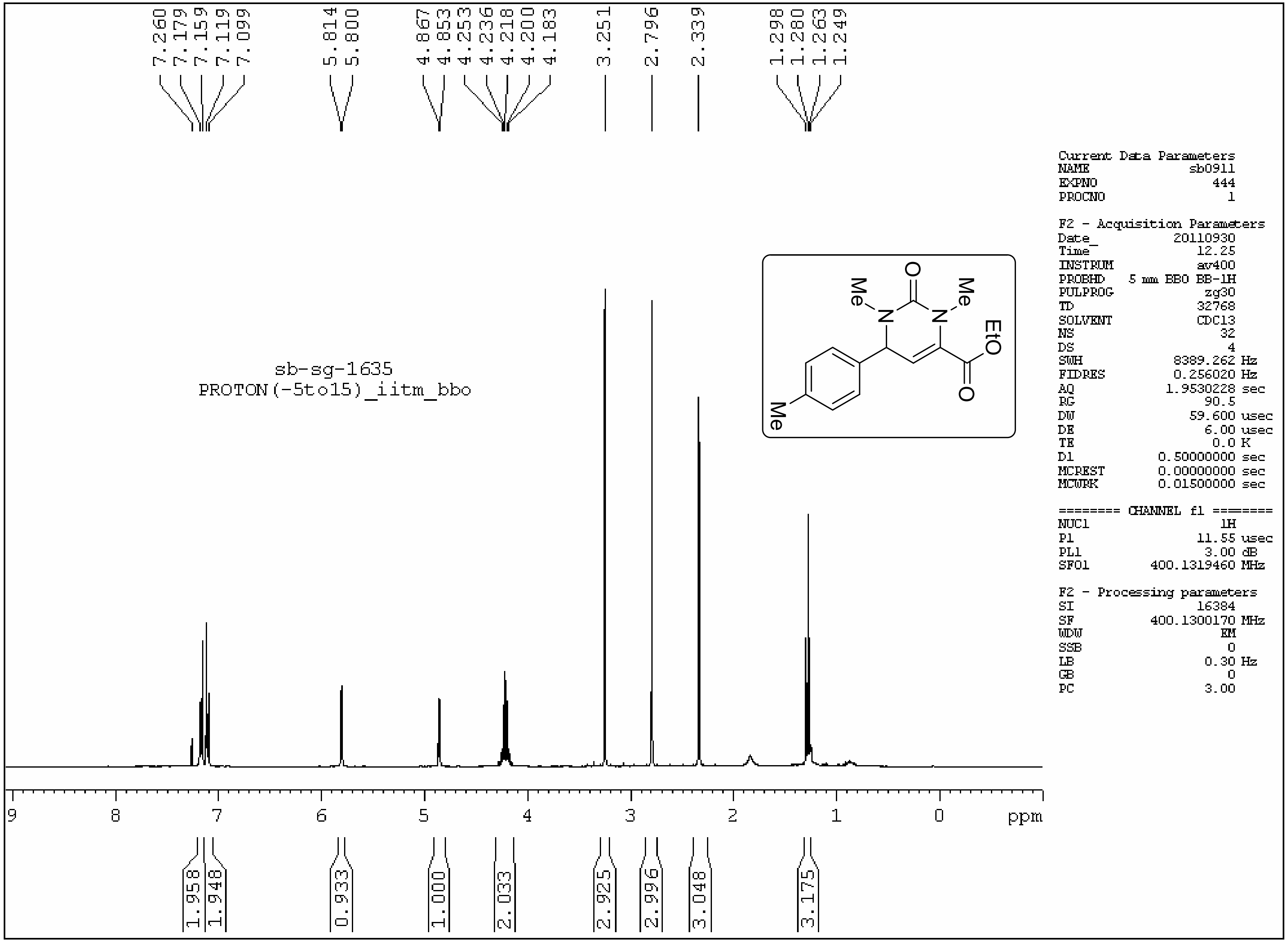

^{1}H NMR spectrum of compound **16**

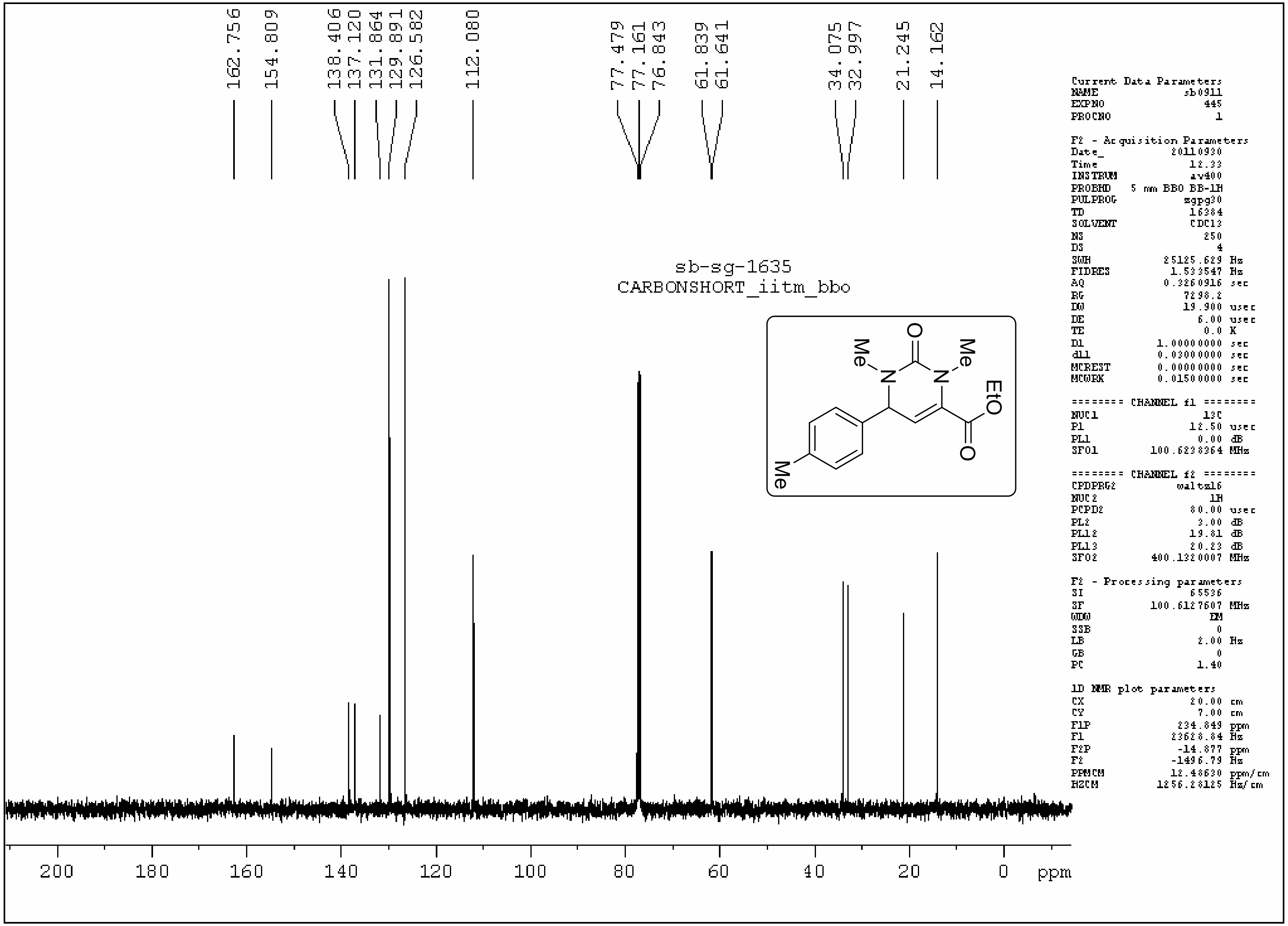

^{13}C NMR spectrum of compound **16**

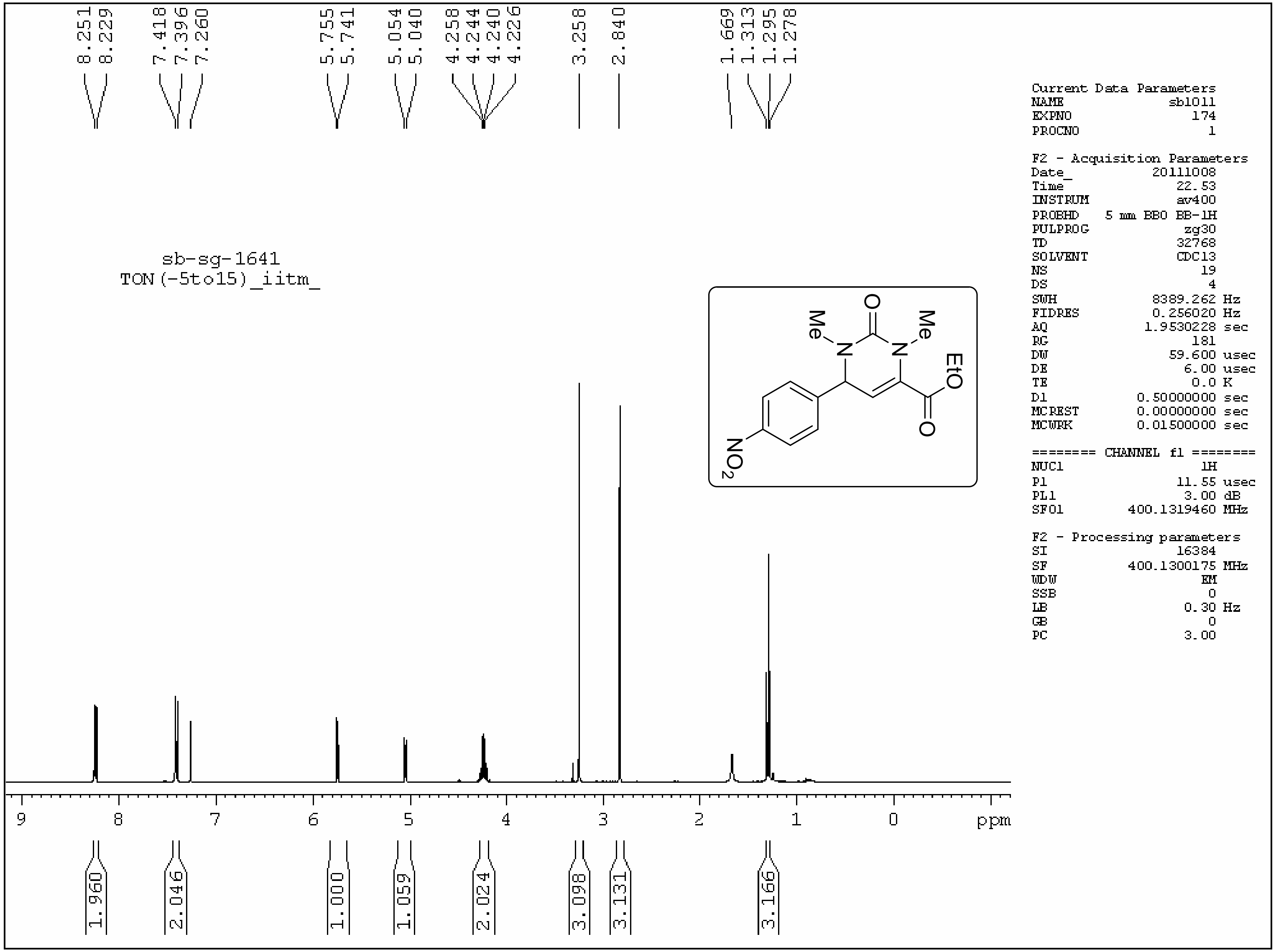

^{1}H NMR spectrum of compound **18**

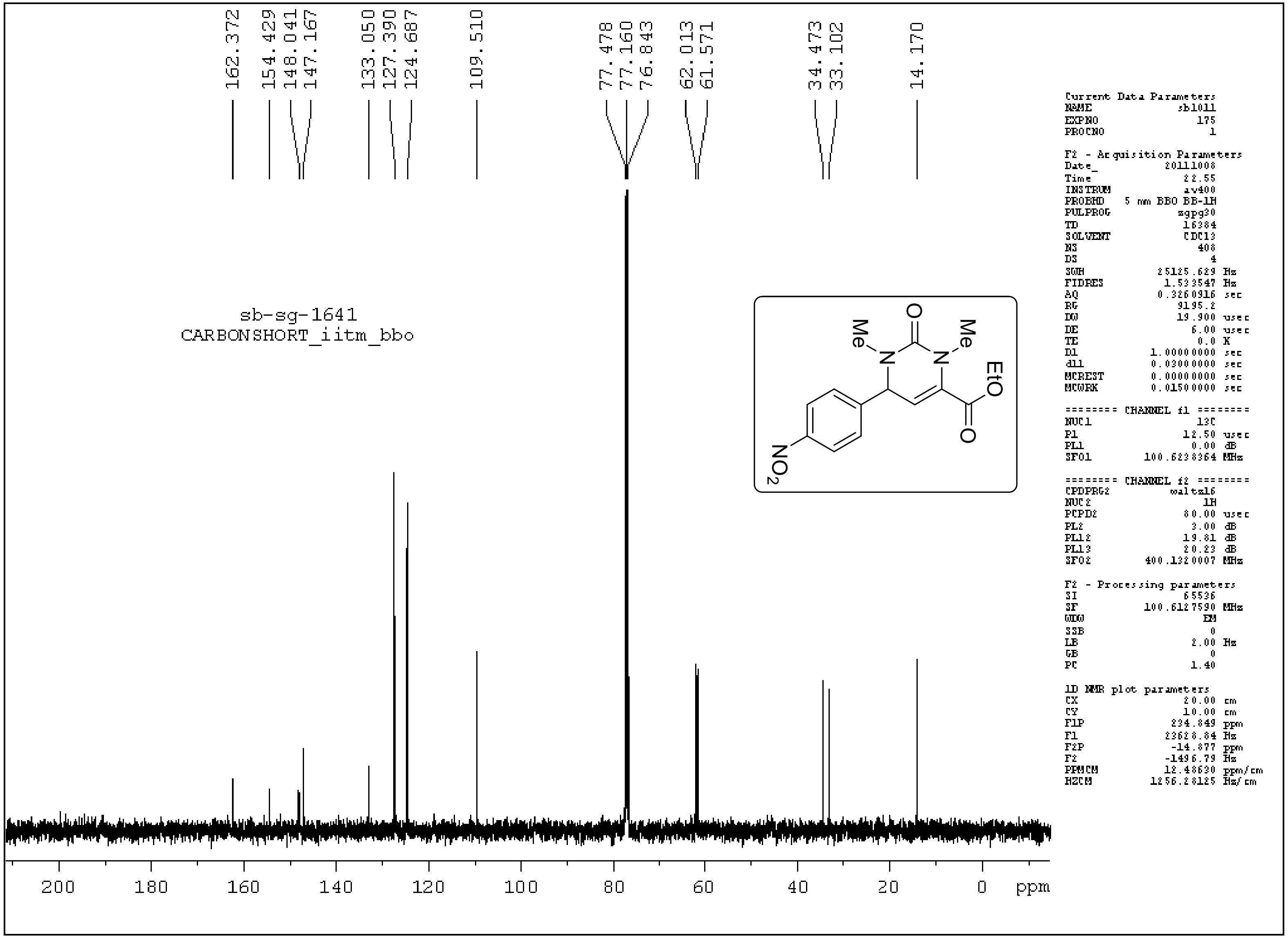

^{13}C NMR spectrum of compound **18**

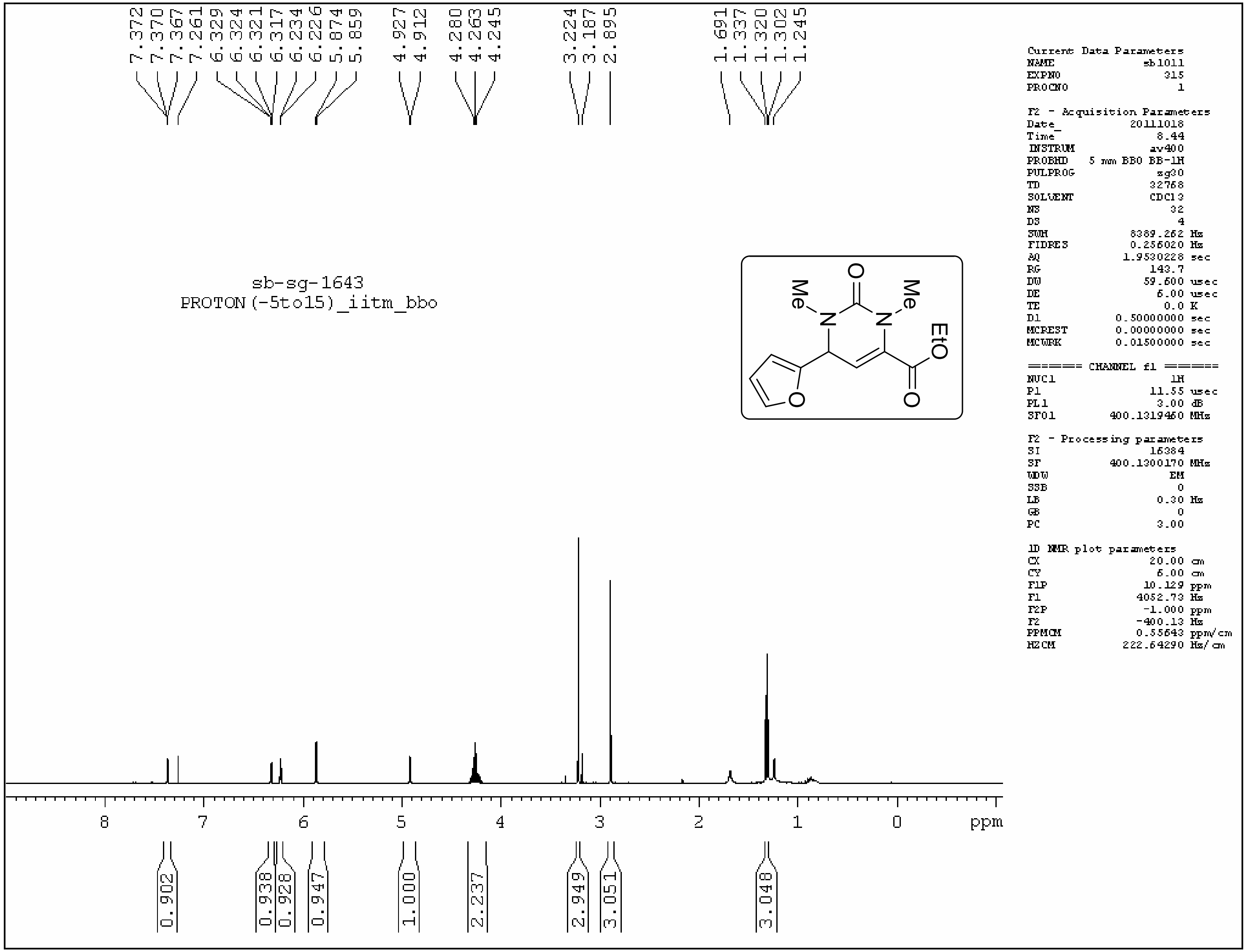

^{1}H NMR spectrum of compound **22**

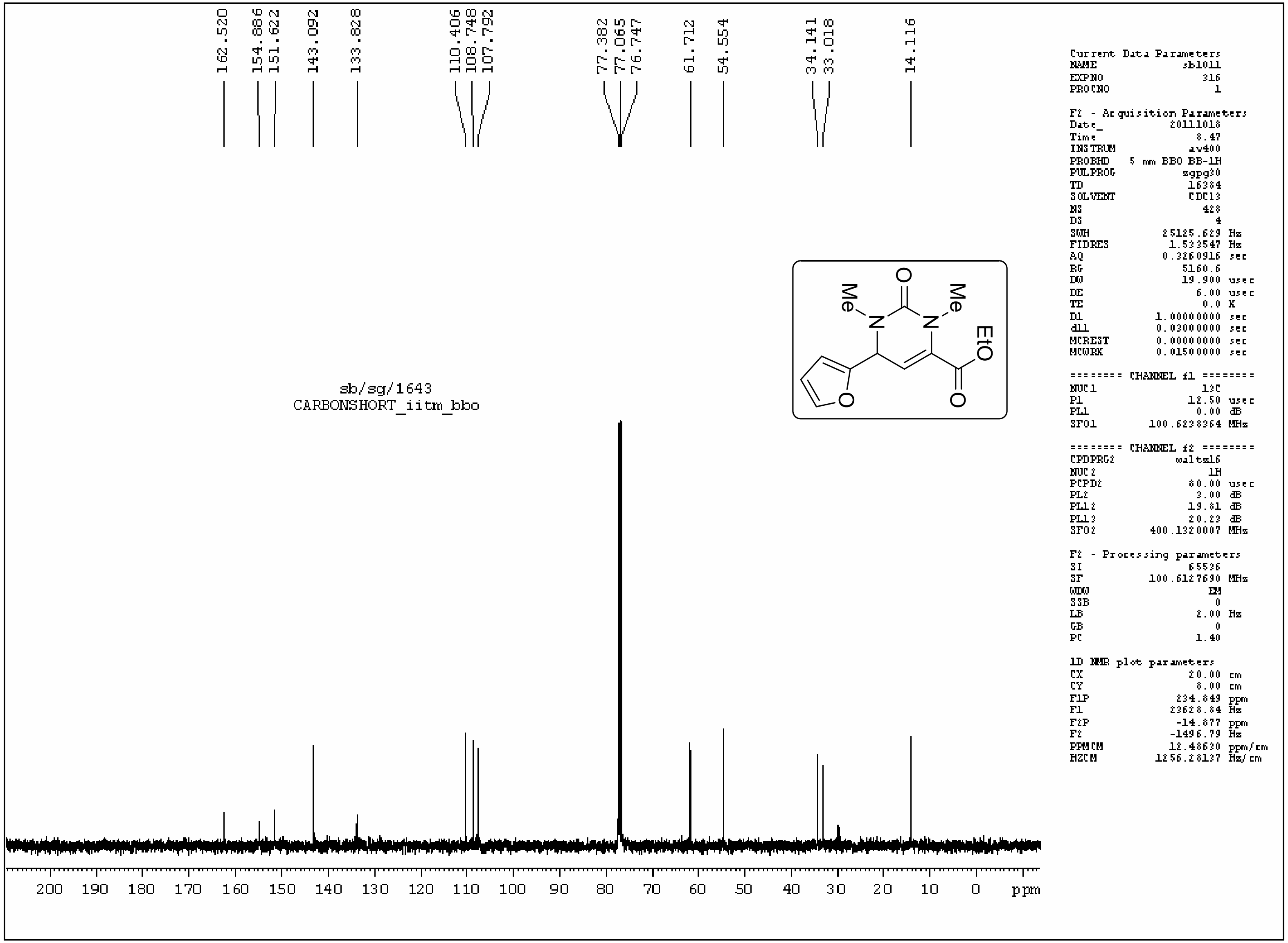

^{13}C NMR spectrum of compound **22**

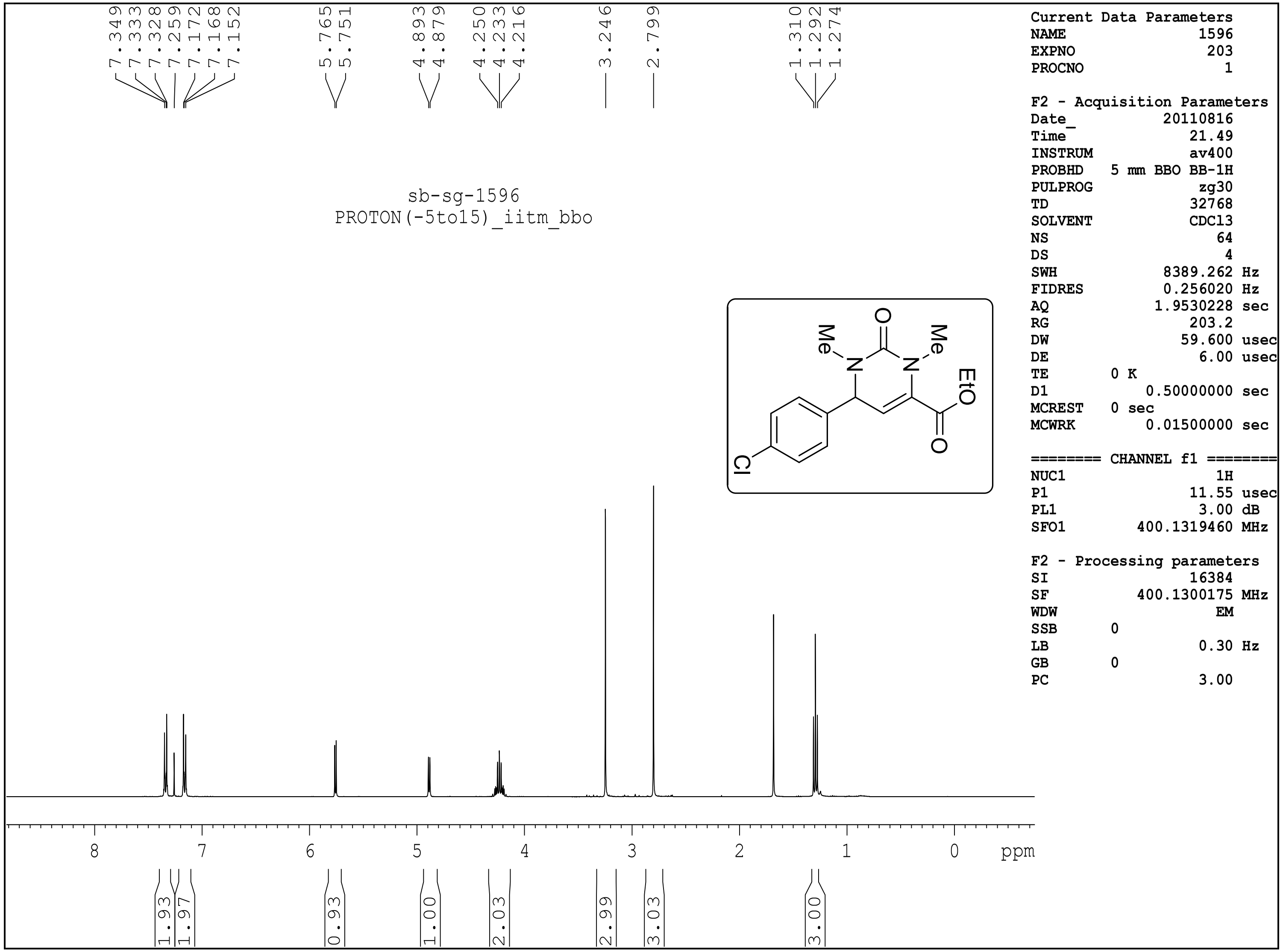

^{1}H NMR spectrum of compound **26**

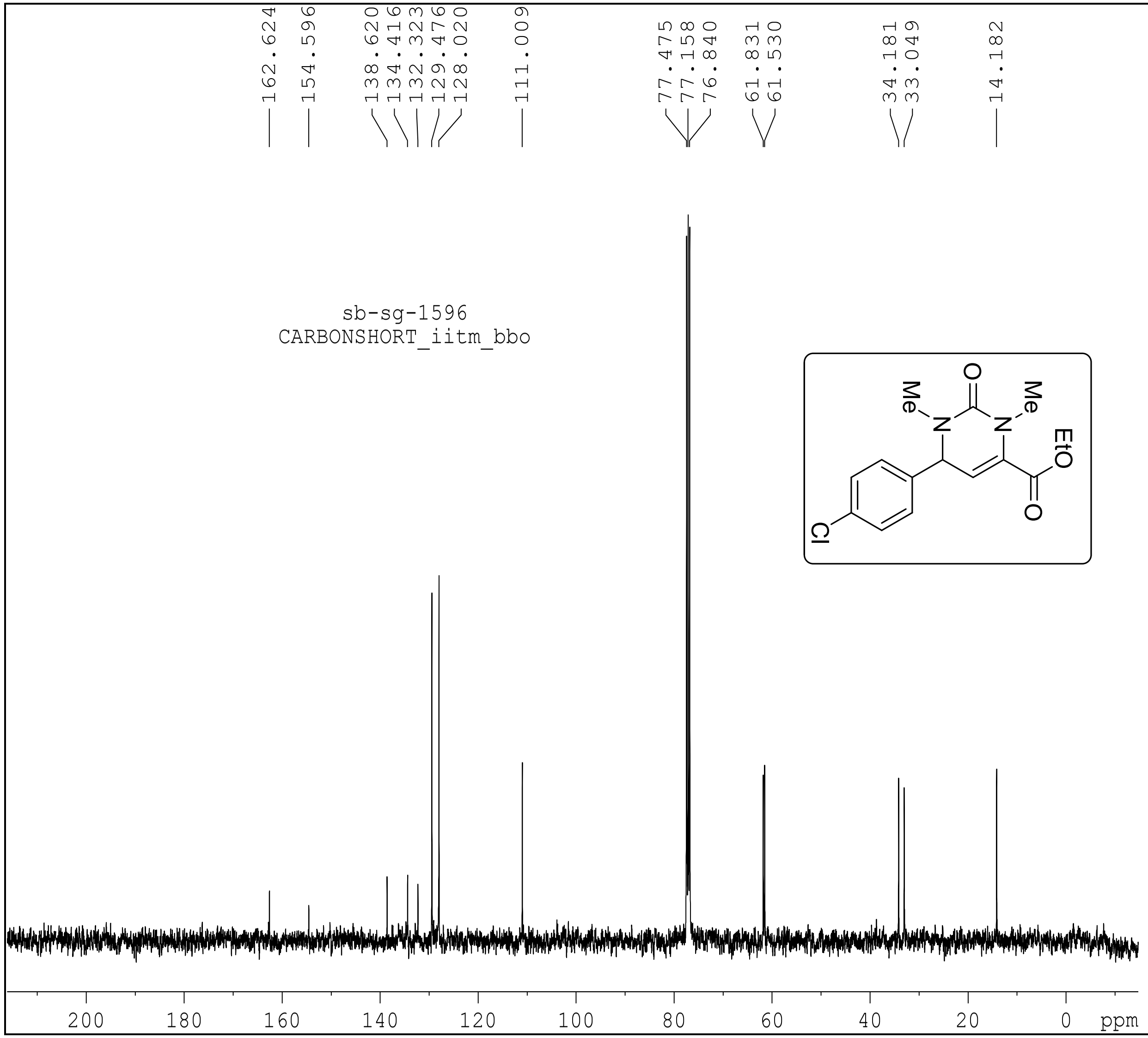

Current Data Parameters	
NAME	1596
EXPNO	204
PROCNO	1
F2 - Acquisition Parameters	
Date_	20110816
Time_	21.59
INSTRUM	av400
PROBHD	5 mm BBO BB-1H
PULPROG	zgpg30
TD	16384
SOLVENT	CDCl3
NS	416
DS	4
SWH	25125.629 Hz
FIDRES	1.533547 Hz
AQ	0.3260916 sec
RG	20642.5
DW	19.900 usec
DE	6.00 usec
TE	0 K
D1	1.00000000 sec
d11	0.03000000 sec
MCREST	0 sec
MCWRK	0.01500000 sec
======== CHANNEL f1 ========	
NUC1	13C
P1	12.50 usec
PL1	0 dB
SFO1	100.6238364 MHz
======== CHANNEL f2 ========	
CPDPRG2	waltz16
NUC2	1H
PCPD2	80.00 usec
PL2	3.00 dB
PL12	19.81 dB
PL13	20.23 dB
SFO2	400.1320007 MHz
F2 - Processing parameters	
SI	65536
SF	100.6127591 MHz
WDW	EM
SSB	0
LB	4.00 Hz
GB	0
PC	1.40

^{13}C NMR spectrum of compound **26**

References

[1] Meng, Q.; Zhu, L.; Zhang, Z. J. Org. Chem. **2008**, *73*, 7209.

[2] Akkari, R., et. al. Substituted pyrazolo[3,4-B]pyridine-6-carboxylic acids and their use. World Patent. WO2017060873A1, April 13, 2017.

[3] Gremaud, L.; Alexakis, A. *Angew. Chem., Int. Ed.* **2012,** *51,* 794.

[4] Halland, N.; Velgaard, T.; Jørgensen, K. A. *J. Org. Chem.* **2003,** *68,* 5067.

[5] Gore, S.; Baskaran S.; König, B. *Green Chem*. **2011**, *13*, 1009.
